# Genomic Analysis of AZD1222 (ChAdOx1) Vaccine Breakthrough Infections in the City of Mumbai

**DOI:** 10.1155/2022/2449068

**Published:** 2022-02-11

**Authors:** Arusha Shetty, Gaurav Chatterjee, Sweta Rajpal, Tuhina Srivastava, Nilesh Gardi, Sumeet Mirgh, Anant Gokarn, Sachin Punatar, Nitin Shetty, Amit Joshi, Sudhir Nair, Vedang Murthy, Navin Khattry, Prashant Tembhare, Rajesh Dikshit, Pankaj Chaturvedi, Ashwini More, Sujeet Kamtalwar, Preeti Chavan, Vivek Bhat, Amar Patil, Sachin Dhumal, Prashant Bhat, Papagudi Subramanian, Sumeet Gujral, Rajendra Badwe, Nikhil Patkar, Sudeep Gupta

**Affiliations:** ^1^Haematopathology Laboratory, ACTREC, Tata Memorial Centre, Navi Mumbai, India; ^2^Homi Bhabha National Institute (HBNI), Mumbai, India; ^3^Department of Medical Oncology, Tata Memorial Centre, Mumbai, India; ^4^Department of Radiodiagnosis, Tata Memorial Centre, Mumbai, India; ^5^Department of Surgical Oncology, Tata Memorial Centre, Mumbai, India; ^6^Department of Radiation Oncology, Tata Memorial Centre, Mumbai, India; ^7^Centre for Cancer Epidemiology, Tata Memorial Centre, Navi Mumbai, India; ^8^Department of General Medicine, ACTREC, Tata Memorial Centre, Navi Mumbai, India; ^9^Department of Laboratory Medicine, ACTREC, Tata Memorial Centre, Navi Mumbai, India; ^10^Department of Microbiology, ACTREC, Tata Memorial Centre, Navi Mumbai, India

## Abstract

**Background:**

This manuscript describes the genetic features of SARS-CoV-2 mutations, prevalent phylogenetic lineages, and the disease severity amongst COVID-19-vaccinated individuals in a tertiary cancer hospital during the second wave of the pandemic in Mumbai, India.

**Methods:**

This observational study included 159 COVID-19 patients during the second wave of the pandemic from 17^th^ March to 1^st^ June 2021 at a tertiary cancer care centre in Mumbai. The cohort comprised of healthcare workers, staff relatives, cancer patients, and patient relatives. For comparison, 700 SARS-CoV-2 genomes sequenced during the first wave (23^rd^ April to 25^th^ September 2020) at the same centre were also analysed. Patients were assigned to nonvaccinated (no vaccination or <14 days from the 1^st^ dose, *n* = 92), dose 1(≥14 days from the 1^st^ dose to <14 days from the 2^nd^ dose, *n* = 29), and dose 2 (≥14 days from the 2^nd^ dose, *n* = 38) groups. Primary measure was the prevalence of SARS-CoV-2 genomic lineages among different groups. In addition, severity of COVID-19 was assessed according to clinical and genomic variables.

**Results:**

Kappa B.1.1671.1 and delta B.1.617.2 variants contributed to an overwhelming majority of sequenced genomes (unvaccinated: 40/92, 43.5% kappa, 46/92, 50% delta; dose 1: 14/29, 48.3% kappa, 15/29, 51.7% delta; and dose 2: 23/38, 60.5% kappa, 14/38 36.8% delta). The proportion of the kappa and delta variants did not differ significantly across the unvaccinated, dose 1, and dose 2 groups (*p* = 0.27). There was no occurrence of severe COVID-19 in the dose 2 group (0/38, 0% vs. 14/121, 11.6%; *p* = 0.02). SARS-CoV-2 genomes from all three severe COVID-19 patients in the vaccinated group belonged to the delta lineage (3/28, 10.7% vs. 0/39, 0.0%, *p* = 0.04).

**Conclusions:**

Sequencing analysis of SARS-COV-2 genomes from Mumbai during the second wave of COVID-19 suggests the prevalence of the kappa B.1.617.1 and the delta B.1.627.2 variants among both vaccinated and unvaccinated individuals. Continued evaluation of genomic sequencing data from breakthrough COVID-19 is necessary for monitoring the properties of evolving variants of concern and formulating appropriate immune response boosting and therapeutic strategies.

## 1. Background

The ongoing pandemic caused by the SARS-CoV-2 virus has globally disrupted the economy and has affected the health of millions. The second wave of the pandemic was particularly devastating, with close to 30 million confirmed cases and over 370,000 documented deaths in India to date [1]. This pandemic has also witnessed the development, regulatory approval, and clinical implementation of the SARS-CoV-2 vaccine at an unprecedented pace [2]. Vaccines are an indispensable tool to bring this pandemic under control. Several trials have found SARS-CoV-2 vaccines safe and efficacious in reducing the spread of infection and decreasing the severity of disease in infected individuals [3–5]. The vaccines that the regulatory bodies in India have approved include AZD1222 (ChAdOx1, Serum Institute of India under licence from AstraZeneca) [5] and BBV152 (Covaxin), an indigenous vaccine developed by Bharat Biotech [6]. Healthcare workers in India have been at the frontlines and were amongst the first to receive vaccination for SARS-CoV-2.

However, as the pandemic evolves, mutations in the SARS-CoV-2 genome continue to accumulate, resulting in novel SARS-CoV-2 strains [7]. The emergence of these mutations is not entirely unexpected as RNA viruses (such as influenza virus) have high mutation rates [2]. The genuine concern here is that the emerging S gene mutations may hamper strategies that target the viral spike protein for vaccine generation [7]. Similarly, the emergence of specific mutations may lead to ineffective immune responses due to the phenomenon of immune escape [8]. These findings have suggested a negative impact on vaccine efficacy, which reportedly varied from 96% to 10%, depending upon the type of vaccine administered [9]. Although it is expected that the COVID-19 vaccine protects the recipient from an infection, breakthrough infections have been described in healthcare workers and other vaccinated individuals from India and other countries [10–13]. However, studies that have described the genetic variants of SARS-CoV-2 breakthrough infections in AZD1222 vaccinated patients are largely lacking.

This manuscript describes the genetic features of SARS-CoV-2 mutations, prevalent phylogenetic clades, and the disease severity amongst COVID-19-vaccinated individuals in a tertiary cancer hospital in India. We also describe the genetic changes in SARS-CoV-2 in the current wave of infection compared to the first wave in India.

## 2. Methods

### 2.1. Study Design

This study enrolled SARS-CoV-2-positive patients during the second wave of the pandemic from 17^th^ March 2021 to 1^st^ June 2021 at a single laboratory in a tertiary cancer care centre in Mumbai, India. The cohort comprised of cancer patients being treated at the hospital, patient relatives, staff members, healthcare workers, or staff relatives. The clinical and demographic details were obtained through in-person communication while taking swabs and put in the Indian Council of Medical Research COVID-19 Data Portal (https://cvstatus.icmr.gov.in/). The follow-up details were collected either from our institutional medical records or telephonically for subjects treated elsewhere.

### 2.2. Collection of Samples and Sample Processing

Nasopharyngeal and oropharyngeal swabs were collected and sent to the laboratory as part of routine SARS-CoV-2 testing in a molecular transport medium (MTM). RNA was extracted, and the presence of SARS-CoV-2 infection confirmed using real-time reverse transcriptase polymerase chain reaction (RT-PCR). Positive samples having a cycle threshold (Ct) of less than 30 (for E/N gene) were further used for amplicon-based enrichment followed by sequencing.

### 2.3. Targeted Viral Whole Genome Sequencing

Sequencing libraries of the RNA were prepared using amplicon-based RNA sequencing. Target gene amplification of the virus was carried out using a modified version of the nCoV-2019/V3 primer pools 1 and 2 of custom-designed tiling primers as described by the ARTIC network initiative yielding an amplicon size of ∼400 bp [14]. Illumina compatible sequencing ready libraries were constructed using the QIAseq FX library kit (Qiagen, Germany) as per the manufacturer's instructions and modified as described by the Artic Consortium. Samples were pooled in equimolar concentration. The denatured libraries were subjected to paired-end sequencing (2 × 250 bp) on a MiSeq (Illumina) desktop sequencer with a targeted depth of 0.42 million reads per sample after quality check.

### 2.4. Genomic Data Analysis

Raw fastq sequences were subjected to quality check, carried out by the FastQC (v.0.11.9) tool. A custom, reproducible nextflow workflow was constructed for further data processing. Once the reads were adapter trimmed by TrimGalore, alignment was carried out by BWA-MEM (v.0.7.17) against the reference genome (GenBank : MN908947.3, RefSeq : NC_045512.2; Wuhan-Hu-1 genome). Samtools (v.1.9) was used for processing SAM files to sorted and indexed BAM files. Low-quality bases and primers were trimmed using iVar (v.1.2). The same tool was also used to detect sequence variants with a minimum variant allele frequency of 0.01 and generate consensus sequences in a fastq format. Nextclade (v0.9.0) was used for assigning our sequences to clade and for mutation calling using recommended parameters [15]. The Nextstrain tool was used for phylogenetic analysis and comparison with a previously deposited dataset of 700 SARS-CoV-2 isolates, generated by our group during the first wave of COVID-19 in Mumbai. The sequences were filtered, aligned, and masked to build a phylogeny tree using IQ-TREE. Lineage was dynamically assigned to query sequences using the phylogenetic assignment named Global Outbreak LINeages (PANGOLIN) on 10^th^ June 2021 [16]. Visualisation of the phylogeny tree based on the lineage and vaccination cohort was performed using Auspice.

### 2.5. Assessment of Vaccination Status and Outcome

Patients were assigned to the following groups according to vaccination status: nonvaccinated (patients who did not receive any vaccination or <14 days from the 1^st^ dose of vaccine to positive PCR reaction), vaccinated with the 1^st^ dose (dose 1, ≥14 days of the first dose of vaccination to <14 days of the second dose of vaccination from positive PCR reaction), and vaccinated with the 2^nd^ dose (dose 2, ≥14 days of the second dose of vaccination from positive PCR reaction). This timeline-dependent category assignment was in line with the published phase 3 results of AZD1222 vaccine [5].

Clinical outcome was evaluated using a WHO 8-point scale. The primary endpoint was development of severe COVID-19 defined as any outcome belonging to WHO score ≥ 5 (death, mechanical ventilation with or without additional organ support, and noninvasive ventilation/high-flow oxygen) prior to day 30 from the initial SARS-CoV-2 PCR positivity. The Clopper–Pearson binomial ‘exact' method was used to calculate 95% confidence interval (CI) of proportions. The chi-square test was used to compare the proportion of patients developing severe COVID-19 in various subgroups. MedCalC and R-version-4.0.1 were used for statistical analysis.

## 3. Results

### 3.1. Patient Characteristics

We stored SARS-CoV-2 isolates from a total of 174 unique patients with COVID-19 from 17^th^ March 2021 to 1^st^ June 2021, which coincided with the second wave of COVID-19 in Mumbai. Out of these 174 samples, the final cohort comprised 159 (91.4%) SARS-CoV-2 genomes which met all of the following criteria: (a) showed PCR amplification, (b) Ct value lower than 30, (c) SARS-CoV-2 genome could be successfully sequenced, and (d) demonstrated acceptable sequencing quality (>95% genome coverage and >21,000 bp reference genome alignment). The clinical and demographic characteristics of the cohort can be seen in Table 1. Notably, a significant subset of patients (29/92, 18.2%, 95% CI 12.6–25.1%) had associated malignancy as our hospital is a tertiary cancer care centre. The median age was 36 (range 6–8) years with a male:female ratio of 1.04. All of the subjects were from the state of Maharashtra; however, they showed varied distribution among municipalities such as Raigad (98/159, 61.6%), Thane (36/159, 22.6%), and Mumbai (24/159, 15.1%), indicating multiple, independent source of SARS-CoV-2 infection.

Out of 159 patients, a total of 38 (23.9%, 95% CI 17.5–31.3%) and 29 (18.2%, 95% CI 12.6–25.1%) patients were assigned to the “vaccinated with the 2^nd^ dose” (dose 2, ≥14 days of the second dose of vaccination from positive PCR reaction) and “vaccinated with the 1^st^ dose” (dose 1, ≥14 days of the first dose of vaccination to <14 days of the second dose of vaccination from positive PCR reaction) category, respectively. All patients in the dose 2 group received the AZD1222 vaccine, while 24 (82.8%) and 5 (17.2%) patients in the dose 1 group received the AZD1222 and the BBV152 vaccine. The median duration (range) between receiving the last dose of vaccine and positive SARS-CoV-2 RT-PCR reaction was 33 (14–57) and 34 (16–81) days for dose 1 and dose 2 categories.

### 3.2. Phylogenetic Analysis

**Figure 1:**
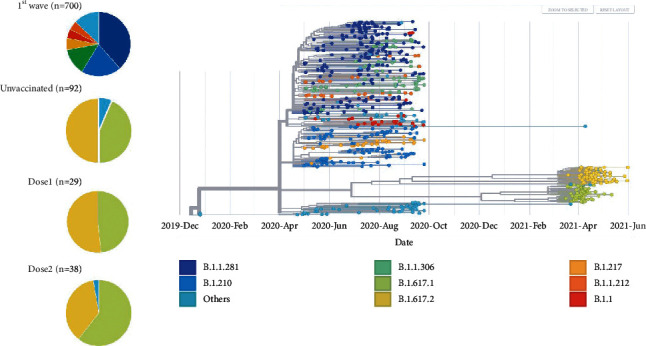
Genomic spectrum of SARS-CoV-2 variants. The phylogenetic tree of 159 SARS-CoV-2 genomes sequenced during the second wave in Mumbai is shown along with the 700 SARS-CoV-2 genomes sequenced during the first wave, colored according to PANGO lineages. Corresponding pie charts show the distribution of prevalent SARS-CoV-2 variants across different groups.

We performed phylogenetic analysis on all 159 SARS-CoV-2 genomes, together with 700 sequences we previously deposited in GISAID during the first wave in Mumbai (April to September, 2020) (Supplementary Table 1) (Figure 1) [17]. The SARS-CoV-2 isolates were assigned to Nextstrain-defined clades and PANGO lineages. An overwhelming majority of the samples belonged to B.1.617 lineages (155/159, 97.5%, 95% CI 93.7–99.3%). Lineage B.1.617.1 (WHO-defined kappa variant, variant of interest, VOI) and Lineage B.1.617.2 (WHO-defined delta variant, variant of concern, VOC) [18] constituted most of the SARS-CoV-2 isolates from unvaccinated (kappa B.1.617.1 40/92, 43.5%, 95% CI 33.2–54.2%; delta B.1.617.2 46/92, 50%, 95% CI 39.4–60.6%), dose 1 (kappa B.1.617.1 14/29, 48.3%, 95% CI 29.5–67.5%; delta B.1.617.2 15/29, 51.7%, 95% CI 32.5–70.6%), and dose 2 (kappa B.1.617.1 23/38, 60.5%, 95% CI 43.4–76.0%; delta B.1.617.2 14/38, 36.8%, 95% CI 21.8–54.0%) groups. The proportion of the predominant lineages B.1.617.2 and B.1.617.1 did not differ significantly across the unvaccinated, dose 1, and dose 2 groups (chi-square *p* = 0.27). Lineage B.1.617.3 (WHO-defined VOI) and lineage B.1.1.7 (WHO-defined alpha variant, VOC) were identified in 3 (1.9%, 95% CI 0.4–5.4%) and 1 patients (0.6%, 95% CI 0.02–3.5%), respectively, belonging to the unvaccinated group. No other WHO-defined VOI or VOC were identified in our dataset.

At the amino acid level, the delta B.1.617.2 variant showed characteristic D614 G, P681 R, L452 R, T19 R, R158 G, 156del, 157del, and T478 K mutations. In addition, 39/75 (52%, 95% CI 40.2–63.7%) and 22/75 (29.3%, 95% CI 19.4–41.0%) of SARS-CoV-2 isolates belonging to lineage B.1.617.2 harbored A222V and D950N substitutions in the S gene. Similarly, kappa B.1.617.1 was characterized by consistent D614G, P681R, L452R, and E484Q mutations in the S gene. Additionally, 53/77 (68.8%, 95% CI 57.3–78.9%), 39/77 (50.7%, 95% CI 39.0–62.2%), 38/77 (49.4%, 37.8–61.0%), 24/77 (31.2%, 95% CI 21.1–42.7%), and 33/77 (42.9%, 95% CI 31.6–54.7%) of SARS-CoV-2 genomes of the B.1.617.1 lineage demonstrated G142D, T95I, Q1071H, E154K, and H1101D spike protein substitutions, respectively. The median number of amino acid substitutions in the cohort was 22 (range 16–27), with a rate of 24.89 substitutions per year.

### 3.3. Assessment of the Clinical Outcome

**Figure 2:**
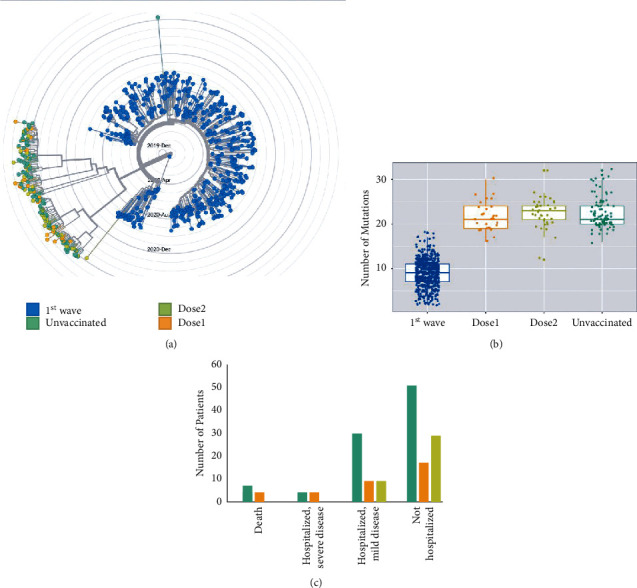
Genomic features and clinical outcome according to groups. (a) The differences in phylogenetic analysis across the various groups. The genomes sequenced at the time of the second wave form distinct clusters as compared to the first wave. The genomes from unvaccinated, dose 1, and dose 2 groups do not show significant differences. (b) Box and dot plots depicting the median number of mutations across different groups. (c) The bar chart showing the distribution of COVID-19 patients according to severity at the time of the second wave of the pandemic.

The clinical outcome of the entire cohort was categorized according to a WHO 8-point scale (Table 1). Overall, 14/159 (8.8%, 95%CI 4.9–14.3%) patients experienced severe COVID-19, defined as any outcome belonging to WHO score ≥ 5. COVID-19 patients with vaccination showed a trend of lower incidence of severe COVID-19 when compared to unvaccinated groups (3/67 (4.5%, 95% CI 0.9–12.5%) vs. 11/92 (12.0%, 95% CI 6.1–20.4%), odds ratio (OR) 0.35, 95% CI 0.09–1.29, *p* = 0.10); however, the association did not reach statistical significance. There was no occurrence of severe COVID-19 in the dose 2 group (0/38, 0% vs. 14/121, 11.6%; *p* = 0.02). Additionally, there was no incidence of mortality in either the dose 1 or dose 2 group. We further analysed the association of various clinical and genomic variables with severity in the entire cohort and vaccinated and unvaccinated cohort separately (Table 2, Supplementary Tables 2 and 3). Biochemical parameters were not assessed for their association with severity as these data were available in only 45 (28.3%) patients. The details are outlined in Table 2 and Figure 2.

In the entire cohort, older age (age ≥ 36 years vs. < 36 years, 15.0% vs. 2.5%, OR 6.79, 95%CI 1.47–31.44, *p* = 0.009) and the presence of associated malignancy (20.6% vs. 6.1%, OR 3.98, 95% CI 1.26–12.54, *p* = 0.02) were significantly associated with a higher incidence of severe COVID-19. No other clinical parameters such as gender, Ct value of E or N gene, or other comorbidities were associated with severe COVID-19 (Table 2). Similarly, the kappa variant (6.4% vs. 12.3%, OR 0.56, 95%CI 0.18–1.76, *p* = 0.41), or delta variant (12.1% vs. 5.8%, OR 2.22, 95%CI 0.71–6.94, *p* = 0.26) did not show a significant association with severe COVID-19. In a multivariable model that included age, sex, malignancy, vaccination status, and genomic variable delta B.1.617.2, only older age (≥36 years) was significantly associated with the incidence of severe COVID-19 (OR 5.71, 95%CI 1.41–28.57, *p* = 0.03).

In the vaccinated group, no clinical variables were associated with a higher incidence of severe COVID-19. SARS-CoV-2 isolates from all of the three severe COVID-19 patients in the vaccinated group belonged to the delta B.1.617.2 lineage (3/28, 10.7%, 95% CI 2.3–28.2%, vs. 0/39, 0.0%, 95% CI 0.0–9.0%, *p* = 0.04). Conversely, no vaccinated patients with the kappa B.1.617.1 lineage (0/37, 0.0%, 95% CI 0.0–9.5%, vs. 3/30, 10.0%, 95% CI 2.1–26.5%, *p* = 0.05) developed severe COVID-19. On analysis of individual amino acid substitutions, mutations associated with the delta variant (T19R, R158G, T478K, and A222V) were associated with a higher incidence of severe COVID-19 among vaccinated patients (Supplementary Table 3).

### 3.4. Comparison of the First Wave with the Second Wave

We further compared the genomic analysis of 159 SARS-CoV-2 isolates during the second wave in Mumbai to our previously deposited dataset of 700 SARS-CoV-2 isolates sequenced during the first wave of COVID-19 in Mumbai (April–September 2020). The most striking finding was the uniform replacement of previously prevalent and more heterogeneous lineages (B.1.1.281, B.1.210, B.1.1.306, B.1.217, B.1.1, B.1.1.212, etc.) by the B.1.617 lineage (Figures 1 and 2). This is consistent with the observation that kappa B.1.617.1 and delta B.1.617.2 lineages originated in India and became the dominant lineages during the second wave. We further observed that there was no occurrence of severe COVID-19 in patients who presented ≥14 days after vaccination with two doses of AZD1222 vaccine (0/38, 0% vs. 14/121, 11.6%; *p* = 0.02).

## 4. Discussion

In this manuscript, we have described the genomic landscape of SARS-CoV-2 isolates during the second wave of COVID-19 (17^th^ March–1^st^ June 2021) in the Mumbai region including a cohort of 67 vaccinated patients. Our findings suggest that the second wave was dominated by the B.1.617.1 kappa and B.1.617.2 delta variants among both vaccinated and unvaccinated COVID-19 patients.

The phenomenon of breakthrough infections in previously vaccinated COVID-19 patients is a matter of concern and interest of global scientific community for its far-reaching implications [11]. Previous studies have found the variant of concern (VOC) as the cause of the overwhelming majority of the breakthrough infections [19]. As of 10^th^ June 2021, four SARS-CoV-2 VOCs are defined by the WHO based on evidence of increased transmissibility, more severe disease, reduced treatment/vaccine efficiency, compromised neutralization by previously generated antibodies, and/or diagnostic detection failure. In addition, few other variants (including kappa B.1.617.1) are classified as variants of interest (VOIs) based on their receptor binding changes, reduced neutralization to antibodies, and predicted increase in transmissibility/disease severity [18]. In particular, the delta variant has been shown to be associated with increased transmissibility and virus infectivity, along with the evidence of immune escape capabilities. The delta variant originated in Maharashtra in late 2020 and rapidly became the dominant lineage of the devastating second wave throughout India [20] and further has spread into >90 countries worldwide. Previous studies on Indian COVID-19 breakthrough infections have identified the delta variant to be the predominant genome in these patients, and the delta variant has been shown to demonstrate higher replication efficiency compared to the alpha variant, greater transmission among health care workers, and reduced sensitivity to antibodies generated by the AZD1222 vaccine [21]. We also found that the kappa and delta variants comprised of almost all of the dose 1 and dose 2 groups, and these were the predominant variants in the unvaccinated group during second wave of COVID-19 in Mumbai as well. We did not observe the K417N mutation characteristic of the AY.1 (delta plus) lineage in any of our sequenced SARS-CoV-2 isolates [22]. Notably, our cohort of dose 1 (29/29, 100%) and dose 2 (36/38, 94.7%) groups were predominantly comprised of symptomatic vaccine breakthrough patients with COVID-19.

We were further able to systematically evaluate the clinical outcome of the entire cohort. Studies evaluating the association of genomic variables with the clinical outcome in COVID-19 patients are scarce [23, 24], particularly in breakthrough infections. Our analysis revealed that older age was associated with a higher incidence of severe COVID-19, in keeping with the published literature [25]. Only 3/67 (4.5%) patients with vaccine breakthrough infections experienced severe COVID-19. However, all of the three patients developing severe COVID-19 among the vaccinated group harbored the delta variant of SARS-CoV-2 (incidence of severe disease 3/28, 10.7%, vs. 0/39, 0.0%, *p* = 0.04). This finding, taken together with the previous demonstration of the properties of the delta variant such as increased transmission among vaccinated healthcare workers and reduced sensitivity to vaccine/disease elicited neutralizing antibodies [20], indicates that long-term COVID-19 appropriate social distancing norms and immunity boosting strategies may be required even in vaccinated individuals with the evolution of fitter viral genomes.

The rapid sharing of genomic sequencing data of SARS-CoV-2 at an unprecedented scale has enabled the scientific community to monitor real-time viral genomic evolution and has been instrumental in identifying new variants [26]. A quick comparison with our previously sequenced 700 SARS-CoV-2 genomes during the first wave of COVID-19 in Mumbai revealed the remarkable dominance of delta and kappa variants during the second wave, as compared to more heterogeneous lineages seen at the time of are scarce first wave. Our data further establish a second benchmark for the prevalence and evolution of SARS-CoV-2 lineages for future genomic sequencing of COVID-19 patients in this region, underscoring the importance of performing and sharing genomic sequencing data during an ongoing pandemic. We acknowledge the limitations of our study that include a heterogeneous sampling strategy, lack of comprehensive epidemiological data, immunoglobulin levels in vaccinated patients and functional analyses, and possibly underpowered evaluation of the association of severe COVID-19 with various subgroups.

## 5. Conclusions

In summary, our results suggest that the kappa B.1.617.1 and the delta B.1.627.2 variants were prevalent among both vaccinated and unvaccinated patients with COVID-19 during the second wave of the pandemic in the Mumbai city region. Continued evaluation of genomic sequencing data from breakthrough COVID-19 is necessary for monitoring properties of evolving variants of concern and formulating appropriate immune response boosting and therapeutic strategies.

## Figures and Tables

**Table 1:** Clinical and demographic details of the cohort (*n* = 159).

	Unvaccinated (*n* = 92)	Vaccinated 1^st^ dose (*n* = 29)	Vaccinated 2^nd^ dose (*n* = 38)
Median age (range)	37.5 (6–77) years	40 (20–81) years	33 (9–62) years
Male:female	1:1	1:9	0 : 6
*Symptomatic (n, %)*	79 (85.9)	29 (100%)	36 (94.7%)
Fever (*n*, %)	60 (65.2%)	24 (82.8%)	20 (52.6%)
Cough (*n*, %)	35 (38%)	13 (44.8%)	17 (44.7%)
Sore throat (*n*, %)	17 (18.5%)	6 (20.7%)	14 (36.8%)
Body ache (*n*, %)	15 (16.3%)	13 (44.8%)	19 (50%)
Breathlessness (*n*, %)	14 (15.2%)	9 (31%)	4 (10.5%)
*Comorbidities (n, %)*	39 (42.4%)	12 (41.4%)	7 (18.4%)
Malignancies (*n*, %)	27 (29.3%)	1 (3.4%)	1 (2.6%)
Diabetes mellitus (n, %)	8 (8.7%)	4 (13.8%)	2 (5.3%)
Hypertension (*n*, %)	7 (7.6%)	5 (17.2%)	3 (7.9%)
*Distribution according to municipality*			
Mumbai	11 (11.9%)	6 (20.7%)	7 (18.4%)
Palghar	1 (1.1%)	0	0
Raigad	57 (61.9%)	19 (65.5%)	22 (57.9%)
Thane	23 (25%)	4 (13.8%)	9 (23.7%)
*Type of vaccine (total, AZD1222:BBV152)*	16 (<14 days after the first dose), 11 : 5	29, 24 : 5	38, 38 : 0
14–20 days from the 1^st^ dose		6 (20.7%)	
21–28 days from the 1^st^ dose		4 (13.8%)	
28+ days from the 1^st^ dose		12 (65.5%)	
14–20 days from the 2^nd^ dose			6 (15.8%)
21–28 days from the 2^nd^ dose			8 (21.1%)
28+ days from the 2^nd^ dose			24 (63.2%)
*SARS-CoV-2 genomic sequencing*			
B.1.617.1	40 (43.5%)	14 (48.3%)	23 (60.5%)
B.1.617.2	46 (50%)	15 (51.7%)	14 (36.8%)
B.1.617.3	3 (3.2%)	0	0
B.1.1.7	1 (1.1%)	0	0
Others	2 (2.2%)	0	1 (2.6%)
*CBC (data available in n* *=* *45)*	(*n* = 31)	(*n* = 9)	(*n* = 5)
Median TLC x 10^9/L (range)	5.23 (0.27–9.04)	5.27 (3.39–14.98)	5.17 (4.68–8.3)
Median worst ALC x 10^9^/L (range)	0.89 (0.11–3.19)	0.88 (0.36–1.2)	0.9 (1.3–1.7)
*Biochemical parameters (data available in n* *=* *45)*	(*n* = 31)	(*n* = 9)	(*n* = 5)
Median worst CRP mg/L (range)	2.15 (0.1–32.0)	1.8 (0.5–16.22)	2.0 (0.2–3.4)
Median highest IL6 pg/mL (range)	7.8 (0.7–8677)	14.3 (0.4–69.2)	8.3 (0.8–16.1)
Median worst procalcitonin ng/mL (range)	0.05 (0.05–151.5)	0.05 (0.05–0.92)	0.05 (0.05–0.05)
Median worst fibrinogen mg/dL (range)	345 (197–539)	361 (248–613)	349 (284–523)
Median worst D-dimer ng/mL (range)	219 (127–878)	200 (148–522)	213 (177–220)
*Distribution according to the clinical outcome*			
Not hospitalized (WHO score 1–2)	51 (55.4%)	17 (58.6%)	29 (76.3%)
Hospitalized, mild disease (WHO score 3–4)	30 (32.6%)	9 (31.0%)	9 (23.7%)
Hospitalized, severe disease (WHO score 5–7)	7 (7.6%)	3 (10.3%)	0
Death (WHO score 8)	4 (4.3%)	0	0

**Table 2:** Distribution of severe COVID-19 according to clinical and genomic variables (*n* = 159, continuous variables were dichotomized based on the median value).

Category	Univariable analysis		Multivariable analysis	
	% severe disease, odds ratio (95% CI)	*p* value	Odds ratio (95% CI)	*p* value
Age (≥36 (*n* = 80) vs. < 36 (*n* = 79) years)	15.0% vs. 2.5%, 6.79 (1.47–31.44)	**0.009**	5.71 (1.41–38.57)	**0.03**
Gender (male (*n* = 81) vs. female (*n* = 78))	11.1% vs. 6.4%, 1.83 (0.58–5.71)	0.40	1.50 (0.46–5.40)	0.51
Ct value of the E gene (<22.5 (*n* = 79) vs. ≥ 22.5 (*n* = 80))	7.5% vs. 10.0%, 0.74 (0.24–2.24)	0.78		
Ct value of the N gene (<23 (*n* = 80) vs. ≥ 23 (*n* = 79))	8.7% vs. 8.8%, 0.99 (0.33–2.95)	0.99		
Total no. of amino acid mutations per sample (≥22 (*n* = 83) vs. < 22 (*n* = 76))	8.4% vs. 9.2%, 0.91 (0.30–2.72)	0.99		
Diabetes (presence (*n* = 14) vs. absence (*n* = 145))	14.2% vs. 8.2%, 1.85 (0.37–9.23)	0.36		
Hypertension (presence (*n* = 15) vs. absence (*n* = 144))	6.6% vs. 9.0%, 0.72 (0.09–5.92)	0.99		
Malignancy (presence (*n* = 29) vs. absence (*n* = 130))	20.6% vs. 6.1%, 3.98 (1.26–12.54)	0.02	1.98 (0.53–7.25)	0.30
Comorbidity (presence of any comorbidity (*n* = 58) vs. absence of all comorbidities (*n* = 101))	12.0% vs. 6.9%, 1.84 (0.61–5.54)	0.38		
Vaccination status (vaccinated (*n* = 67) vs. unvaccinated (*n* = 92))	4.5% vs. 12.0% 0.35 (0.09–1.29)	0.10	0.45 (0.09–1.82)	0.28

*PANGOLIN lineages*				
Kappa B.1.617.1 (presence (*n* = 77) vs. absence (*n* = 82))	6.4% vs. 12.3%, 0.56 (0.18–1.76)	0.41		
Delta B.1.617.2 (presence (*n* = 74) vs. absence (*n* = 85))	12.1% vs. 5.8%, 2.22 (0.71–6.94)	0.26	2.17 (0.68–7.70)	0.20

*Spike protein mutations*				
D614G (presence (*n* = 158) vs. absence (*n* = 1))	8.8% vs. 0.0%, (-)	0.99		
P681R (presence (*n* = 157) vs. absence (*n* = 2))	8.9% vs. 0.0%, (-)	0.99		
L452R (presence (*n* = 157) vs. absence (*n* = 2))	8.9% vs. 0.0%, (-)	0.99		
E484Q (presence (*n* = 80) vs. absence (*n* = 79))	6.2% vs. 11.3%, 0.52 (0.17–1.62)	0.28		
T19R (presence (*n* = 79) vs. absence (*n* = 80))	11.3% vs. 6.25%, 1.93 (0.62–6.03)	0.28		
R158G (presence (*n* = 76) vs. absence (*n* = 83))	10.5% vs. 7.2%, 1.51 (0.50–4.57)	0.58		
T478K (presence (*n* = 76) vs. absence (*n* = 83))	11.8% vs. 6.02%, 2.10 (0.67–6.56)	0.26		
G142D (presence (*n* = 54) vs. absence (*n* = 105))	3.7% vs. 11.4%, 0.30 (0.06–1.38)	0.14		
T95I (presence (*n* = 49) vs. absence (*n* = 110))	8.1% vs. 9.0%, 0.89 (0.26–2.99)	0.99		
A222V (presence (*n* = 41) vs. absence (*n* = 118))	17.0% vs. 5.9%, 3.26 (1.07–9.96)	0.05		
Q1071H (presence (*n* = 38) vs. absence (*n* = 121))	7.8% vs. 9.0%, 0.86 (0.23–3.25)	0.82		
H1101D (presence (*n* = 33) vs. absence (*n* = 126))	3.0% vs. 10.3%, 0.27 (0.03–2.16)	0.30		
D950N (presence (*n* = 26) vs. absence (*n* = 133))	11.5% vs. 8.2%, 1.45 (0.37–5.59)	0.70		
E154K (presence (*n* = 24) vs. absence (*n* = 135))	12.5% vs. 8.1%, 1.61 (0.41–6.26)	0.45		
Q677H (presence (*n* = 10) vs. absence (*n* = 149))	20% vs. 8.0%, 2.85 (0.54–14.98)	0.22		

## Data Availability

All the 159 SARS-CoV-2 genomes sequenced in this study along with the 700 SARS-CoV-2 genomes sequenced at the time of the first wave have been deposited in GISAID (https://www.gisaid.org/). The details with GSAID ID are provided in Supplementary Table 1.

## Supplementary Materials

Supplementary Materials Supplementary Table 1: details along with GISAID ID of all sequenced SARS-CoV-2 genomes in this study. Supplementary Table 2: distribution of severe COVID-19 among the unvaccinated (*n* = 92) patients according to clinical and genomic variables (continuous variables were dichotomized based on the median value in the entire cohort). Supplementary Table 3: distribution of severe COVID-19 among the vaccinated (*n* = 67) patients according to clinical and genomic variables (continuous variables were dichotomized based on the median value in the entire cohort).
