# Supplementary material for: Genomic Analysis of AZD1222 (ChAdOx1) Vaccine Breakthrough Infections in the City of Mumbai
Source: Int J Clin Pract. 2022 Feb 11;2022:2449068. doi: 10.1155/2022/2449068 (PMC9159196; doi:10.1155/2022/2449068)
Supplement: Supplementary Materials — Supplementary Table 1: details along with GISAID ID of all sequenced SARS-CoV-2 genomes in this study. Supplementary Table 2: distribution of severe COVID-19 among the unvaccinated (n = 92) patients according to clinical and genomic variables (continuous variables were dichotomized based on the median value in the entire cohort). Supplementary Table 3: distribution of severe COVID-19 among the vaccinated (n = 67) patients according to clinical and genomic variables (continuous variables were dichotomized based on the median value in the entire cohort). [file 2449068.f1.zip › 2449068.f1/Supplementary Table 1.docx]

**Supplementary Table 1:** Details along with GISAID ID of all sequenced SARS-CoV-2 genomes in this study

| **STRAIN** | **VIRUS** | **GISAID_EPI_ISL** | **DATE_SEQUENCED** | **COUNTRY** | **AGE** | **SEX** | **PANGOLIN_LINEAGE** | **WAVE OF COVID-19 PANDEMIC IN INDIA** |
| --- | --- | --- | --- | --- | --- | --- | --- | --- |
| hCoV-19/India/UP-ACTREC-001/2020 | betacoronavirus | EPI_ISL_699658 | 23-04-2020 | India | 57 | Female | B.6 | First |
| hCoV-19/India/MH-ACTREC-002/2020 | betacoronavirus | EPI_ISL_699659 | 29-04-2020 | India | 33 | Female | B.6 | First |
| hCoV-19/India/MH-ACTREC-003/2020 | betacoronavirus | EPI_ISL_699660 | 01-05-2020 | India | 32 | Female | B.1.210 | First |
| hCoV-19/India/MH-ACTREC-004/2020 | betacoronavirus | EPI_ISL_699661 | 01-05-2020 | India | 32 | Female | B.1 | First |
| hCoV-19/India/MH-ACTREC-005/2020 | betacoronavirus | EPI_ISL_699662 | 01-05-2020 | India | 39 | Male | B.1.1.281 | First |
| hCoV-19/India/MH-ACTREC-006/2020 | betacoronavirus | EPI_ISL_699663 | 02-05-2020 | India | 61 | Male | B.1.1.281 | First |
| hCoV-19/India/MH-ACTREC-007/2020 | betacoronavirus | EPI_ISL_699664 | 02-05-2020 | India | 11 | Female | B.1 | First |
| hCoV-19/India/MH-ACTREC-008/2020 | betacoronavirus | EPI_ISL_699665 | 02-05-2020 | India | 4 | Male | B.1.1.281 | First |
| hCoV-19/India/MH-ACTREC-009/2020 | betacoronavirus | EPI_ISL_699666 | 02-05-2020 | India | 2 | Male | B.1.1.281 | First |
| hCoV-19/India/MH-ACTREC-010/2020 | betacoronavirus | EPI_ISL_699667 | 02-05-2020 | India | 34 | Male | B.1.1.281 | First |
| hCoV-19/India/MH-ACTREC-011/2020 | betacoronavirus | EPI_ISL_699668 | 02-05-2020 | India | 50 | Male | B.1.1.281 | First |
| hCoV-19/India/MH-ACTREC-012/2020 | betacoronavirus | EPI_ISL_723045 | 02-05-2020 | India | 48 | Female | B.1.217 | First |
| hCoV-19/India/MH-ACTREC-013/2020 | betacoronavirus | EPI_ISL_699669 | 02-05-2020 | India | 42 | Female | B.1.1.281 | First |
| hCoV-19/India/MH-ACTREC-014/2020 | betacoronavirus | EPI_ISL_699670 | 03-05-2020 | India | 29 | Female | B.1.1.306 | First |
| hCoV-19/India/MH-ACTREC-015/2020 | betacoronavirus | EPI_ISL_699671 | 03-05-2020 | India | 25 | Female | B.1.1.306 | First |
| hCoV-19/India/MH-ACTREC-016/2020 | betacoronavirus | EPI_ISL_699672 | 03-05-2020 | India | 54 | Male | B.1.1.281 | First |
| hCoV-19/India/MH-ACTREC-017/2020 | betacoronavirus | EPI_ISL_699673 | 03-05-2020 | India | 35 | Male | B.6 | First |
| hCoV-19/India/MH-ACTREC-018/2020 | betacoronavirus | EPI_ISL_699674 | 03-05-2020 | India | 31 | Female | B.1.1.212 | First |
| hCoV-19/India/MH-ACTREC-019/2020 | betacoronavirus | EPI_ISL_699675 | 03-05-2020 | India | 5 | Female | B.1.1.212 | First |
| hCoV-19/India/MH-ACTREC-020/2020 | betacoronavirus | EPI_ISL_699676 | 03-05-2020 | India | 1 | Male | B.1.1.212 | First |
| hCoV-19/India/MH-ACTREC-021/2020 | betacoronavirus | EPI_ISL_699677 | 04-05-2020 | India | 3 | Female | B.1.1.306 | First |
| hCoV-19/India/MH-ACTREC-022/2020 | betacoronavirus | EPI_ISL_699678 | 03-05-2020 | India | 45 | Female | B.1.1.281 | First |
| hCoV-19/India/MH-ACTREC-023/2020 | betacoronavirus | EPI_ISL_699679 | 03-05-2020 | India | 21 | Male | B.1.1.281 | First |
| hCoV-19/India/MH-ACTREC-024/2020 | betacoronavirus | EPI_ISL_699680 | 03-05-2020 | India | 20 | Male | B.1.1.101 | First |
| hCoV-19/India/MH-ACTREC-025/2020 | betacoronavirus | EPI_ISL_699681 | 03-05-2020 | India | 65 | Male | B.1.1.101 | First |
| hCoV-19/India/MH-ACTREC-026/2020 | betacoronavirus | EPI_ISL_699682 | 04-05-2020 | India | 40 | Female | B.1.1.281 | First |
| hCoV-19/India/MH-ACTREC-027/2020 | betacoronavirus | EPI_ISL_699683 | 04-05-2020 | India | 52 | Male | B.1.210 | First |
| hCoV-19/India/MH-ACTREC-028/2020 | betacoronavirus | EPI_ISL_699684 | 04-05-2020 | India | 25 | Male | B.1.1.212 | First |
| hCoV-19/India/MH-ACTREC-029/2020 | betacoronavirus | EPI_ISL_699685 | 05-05-2020 | India | 41 | Male | B.1.1.281 | First |
| hCoV-19/India/MH-ACTREC-030/2020 | betacoronavirus | EPI_ISL_699686 | 06-05-2020 | India | 28 | Male | B.1.1.212 | First |
| hCoV-19/India/MH-ACTREC-031/2020 | betacoronavirus | EPI_ISL_699687 | 06-05-2020 | India | 64 | Male | B.6 | First |
| hCoV-19/India/MH-ACTREC-032/2020 | betacoronavirus | EPI_ISL_699688 | 07-05-2020 | India | 24 | Female | B.1.1.212 | First |
| hCoV-19/India/MH-ACTREC-033/2020 | betacoronavirus | EPI_ISL_699689 | 07-05-2020 | India | 28 | Female | B.1.210 | First |
| hCoV-19/India/MH-ACTREC-034/2020 | betacoronavirus | EPI_ISL_699690 | 07-05-2020 | India | 1 | Female | B.1.210 | First |
| hCoV-19/India/MH-ACTREC-035/2020 | betacoronavirus | EPI_ISL_699691 | 08-05-2020 | India | 43 | Female | B.1.210 | First |
| hCoV-19/India/MH-ACTREC-036/2020 | betacoronavirus | EPI_ISL_699692 | 08-05-2020 | India | 45 | Male | B.1.1.281 | First |
| hCoV-19/India/MH-ACTREC-037/2020 | betacoronavirus | EPI_ISL_699693 | 08-05-2020 | India | 16 | Male | B.1.1.306 | First |
| hCoV-19/India/MH-ACTREC-038/2020 | betacoronavirus | EPI_ISL_699694 | 09-05-2020 | India | 20 | Male | B.1.1.281 | First |
| hCoV-19/India/MH-ACTREC-039/2020 | betacoronavirus | EPI_ISL_699695 | 10-05-2020 | India | 34 | Female | B.1.1.281 | First |
| hCoV-19/India/MH-ACTREC-040/2020 | betacoronavirus | EPI_ISL_699696 | 10-05-2020 | India | 30 | Male | B.1.217 | First |
| hCoV-19/India/MH-ACTREC-041/2020 | betacoronavirus | EPI_ISL_699697 | 11-05-2020 | India | 47 | Female | B.1.1.306 | First |
| hCoV-19/India/MH-ACTREC-042/2020 | betacoronavirus | EPI_ISL_699698 | 12-05-2020 | India | 51 | Male | B.1.210 | First |
| hCoV-19/India/MH-ACTREC-043/2020 | betacoronavirus | EPI_ISL_699699 | 12-05-2020 | India | 53 | Male | B.1.210 | First |
| hCoV-19/India/MH-ACTREC-044/2020 | betacoronavirus | EPI_ISL_699700 | 12-05-2020 | India | 34 | Female | B.1.1.281 | First |
| hCoV-19/India/MH-ACTREC-045/2020 | betacoronavirus | EPI_ISL_699701 | 12-05-2020 | India | 32 | Female | B.1.217 | First |
| hCoV-19/India/MH-ACTREC-046/2020 | betacoronavirus | EPI_ISL_699702 | 13-05-2020 | India | 47 | Male | B.1.1.306 | First |
| hCoV-19/India/MH-ACTREC-047/2020 | betacoronavirus | EPI_ISL_699703 | 13-05-2020 | India | 25 | Male | B.1.210 | First |
| hCoV-19/India/MH-ACTREC-048/2020 | betacoronavirus | EPI_ISL_699704 | 13-05-2020 | India | 45 | Male | B.1.210 | First |
| hCoV-19/India/MH-ACTREC-049/2020 | betacoronavirus | EPI_ISL_699705 | 13-05-2020 | India | 52 | Male | B.1.210 | First |
| hCoV-19/India/MH-ACTREC-050/2020 | betacoronavirus | EPI_ISL_699706 | 13-05-2020 | India | 23 | Male | B.1.210 | First |
| hCoV-19/India/MH-ACTREC-051/2020 | betacoronavirus | EPI_ISL_699707 | 13-05-2020 | India | 37 | Male | B.1.210 | First |
| hCoV-19/India/MH-ACTREC-052/2020 | betacoronavirus | EPI_ISL_699708 | 13-05-2020 | India | 37 | Female | B.1.1.281 | First |
| hCoV-19/India/MH-ACTREC-053/2020 | betacoronavirus | EPI_ISL_699709 | 13-05-2020 | India | 52 | Male | B.1.1.281 | First |
| hCoV-19/India/MH-ACTREC-054/2020 | betacoronavirus | EPI_ISL_699710 | 14-05-2020 | India | 31 | Female | B.1.1.212 | First |
| hCoV-19/India/MH-ACTREC-055/2020 | betacoronavirus | EPI_ISL_699711 | 14-05-2020 | India | 15 | Female | B.1.1.306 | First |
| hCoV-19/India/MH-ACTREC-056/2020 | betacoronavirus | EPI_ISL_699712 | 14-05-2020 | India | 33 | Female | B.1.210 | First |
| hCoV-19/India/MH-ACTREC-057/2020 | betacoronavirus | EPI_ISL_699713 | 14-05-2020 | India | 33 | Female | B.1.210 | First |
| hCoV-19/India/MH-ACTREC-058/2020 | betacoronavirus | EPI_ISL_699714 | 15-05-2020 | India | 59 | Male | B.1.1 | First |
| hCoV-19/India/MH-ACTREC-059/2020 | betacoronavirus | EPI_ISL_699715 | 15-05-2020 | India | 41 | Male | B.1.5 | First |
| hCoV-19/India/MH-ACTREC-060/2020 | betacoronavirus | EPI_ISL_699716 | 16-05-2020 | India | 30 | Female | B.1.1.281 | First |
| hCoV-19/India/MH-ACTREC-061/2020 | betacoronavirus | EPI_ISL_699717 | 16-05-2020 | India | 71 | Female | B.1.1.281 | First |
| hCoV-19/India/MH-ACTREC-062/2020 | betacoronavirus | EPI_ISL_699718 | 16-05-2020 | India | 50 | Female | B.1.247 | First |
| hCoV-19/India/MH-ACTREC-063/2020 | betacoronavirus | EPI_ISL_699719 | 17-05-2020 | India | 29 | Female | B.1.5 | First |
| hCoV-19/India/MH-ACTREC-064/2020 | betacoronavirus | EPI_ISL_699720 | 17-05-2020 | India | 7 | Male | B.1.5 | First |
| hCoV-19/India/MH-ACTREC-065/2020 | betacoronavirus | EPI_ISL_699721 | 17-05-2020 | India | 50 | Male | B.1.210 | First |
| hCoV-19/India/MH-ACTREC-066/2020 | betacoronavirus | EPI_ISL_699722 | 17-05-2020 | India | 32 | Female | B.1.210 | First |
| hCoV-19/India/MH-ACTREC-067/2020 | betacoronavirus | EPI_ISL_699723 | 06-05-2020 | India | 40 | Female | B.1.1.281 | First |
| hCoV-19/India/MH-ACTREC-068/2020 | betacoronavirus | EPI_ISL_699724 | 17-05-2020 | India | 28 | Male | B.1.1.306 | First |
| hCoV-19/India/MH-ACTREC-069/2020 | betacoronavirus | EPI_ISL_699725 | 17-05-2020 | India | 31 | Male | B.1.1.281 | First |
| hCoV-19/India/MH-ACTREC-070/2020 | betacoronavirus | EPI_ISL_699726 | 17-05-2020 | India | 20 | Male | B.1.210 | First |
| hCoV-19/India/MH-ACTREC-071/2020 | betacoronavirus | EPI_ISL_699727 | 18-05-2020 | India | 10 | Male | B.1.1.101 | First |
| hCoV-19/India/MH-ACTREC-072/2020 | betacoronavirus | EPI_ISL_699728 | 18-05-2020 | India | 10 | Male | B.1.1.101 | First |
| hCoV-19/India/MH-ACTREC-073/2020 | betacoronavirus | EPI_ISL_699729 | 18-05-2020 | India | 40 | Male | B.1.1.281 | First |
| hCoV-19/India/MH-ACTREC-074/2020 | betacoronavirus | EPI_ISL_699730 | 18-05-2020 | India | 56 | Female | B.1.1.281 | First |
| hCoV-19/India/MH-ACTREC-075/2020 | betacoronavirus | EPI_ISL_699731 | 18-05-2020 | India | 46 | Male | B.1.210 | First |
| hCoV-19/India/WB-ACTREC-076/2020 | betacoronavirus | EPI_ISL_699732 | 18-05-2020 | India | 40 | Female | B.1.1.281 | First |
| hCoV-19/India/MH-ACTREC-077/2020 | betacoronavirus | EPI_ISL_699733 | 18-05-2020 | India | 34 | Female | B.1.210 | First |
| hCoV-19/India/MH-ACTREC-078/2020 | betacoronavirus | EPI_ISL_699734 | 18-05-2020 | India | 36 | Male | B.1.1.281 | First |
| hCoV-19/India/MH-ACTREC-079/2020 | betacoronavirus | EPI_ISL_699735 | 18-05-2020 | India | 40 | Male | B.1.1.281 | First |
| hCoV-19/India/MH-ACTREC-080/2020 | betacoronavirus | EPI_ISL_699736 | 20-05-2020 | India | 50 | Female | B.1.1.212 | First |
| hCoV-19/India/MH-ACTREC-081/2020 | betacoronavirus | EPI_ISL_699737 | 21-05-2020 | India | 68 | Male | B.1.1.281 | First |
| hCoV-19/India/MH-ACTREC-082/2020 | betacoronavirus | EPI_ISL_699738 | 21-05-2020 | India | 48 | Female | B.1.1.281 | First |
| hCoV-19/India/MH-ACTREC-083/2020 | betacoronavirus | EPI_ISL_699739 | 21-05-2020 | India | 33 | Female | B.1.210 | First |
| hCoV-19/India/MH-ACTREC-084/2020 | betacoronavirus | EPI_ISL_699740 | 21-05-2020 | India | 38 | Male | B.1.210 | First |
| hCoV-19/India/MH-ACTREC-085/2020 | betacoronavirus | EPI_ISL_699741 | 21-05-2020 | India | 56 | Male | B.1.1.281 | First |
| hCoV-19/India/MH-ACTREC-086/2020 | betacoronavirus | EPI_ISL_699742 | 21-05-2020 | India | 23 | Male | B.1.1.306 | First |
| hCoV-19/India/MH-ACTREC-087/2020 | betacoronavirus | EPI_ISL_699743 | 21-05-2020 | India | 35 | Female | B.1.1.281 | First |
| hCoV-19/India/MH-ACTREC-088/2020 | betacoronavirus | EPI_ISL_699744 | 21-05-2020 | India | 27 | Male | B.1.210 | First |
| hCoV-19/India/MH-ACTREC-089/2020 | betacoronavirus | EPI_ISL_699745 | 22-05-2020 | India | 55 | Male | B.1.210 | First |
| hCoV-19/India/MH-ACTREC-090/2020 | betacoronavirus | EPI_ISL_699746 | 22-05-2020 | India | 23 | Female | B.1.1.306 | First |
| hCoV-19/India/MH-ACTREC-091/2020 | betacoronavirus | EPI_ISL_699747 | 22-05-2020 | India | 63 | Female | B.1.1.212 | First |
| hCoV-19/India/MH-ACTREC-092/2020 | betacoronavirus | EPI_ISL_699748 | 22-05-2020 | India | 22 | Female | B.1.1.212 | First |
| hCoV-19/India/MH-ACTREC-093/2020 | betacoronavirus | EPI_ISL_699749 | 22-05-2020 | India | 75 | Male | B.1.1.306 | First |
| hCoV-19/India/MH-ACTREC-094/2020 | betacoronavirus | EPI_ISL_699750 | 22-05-2020 | India | 36 | Male | B.1.247 | First |
| hCoV-19/India/MH-ACTREC-095/2020 | betacoronavirus | EPI_ISL_699751 | 22-05-2020 | India | 64 | Female | B.1.1.281 | First |
| hCoV-19/India/MH-ACTREC-096/2020 | betacoronavirus | EPI_ISL_699752 | 22-05-2020 | India | 28 | Male | B.1.210 | First |
| hCoV-19/India/MH-ACTREC-097/2020 | betacoronavirus | EPI_ISL_699753 | 23-05-2020 | India | 45 | Female | B.1.210 | First |
| hCoV-19/India/MH-ACTREC-098/2020 | betacoronavirus | EPI_ISL_699754 | 23-05-2020 | India | 3 months | Female | B.1.1.281 | First |
| hCoV-19/India/MH-ACTREC-099/2020 | betacoronavirus | EPI_ISL_699755 | 23-05-2020 | India | 55 | Male | B.1.1.281 | First |
| hCoV-19/India/MH-ACTREC-100/2020 | betacoronavirus | EPI_ISL_699756 | 23-05-2020 | India | 3 | Female | B.1.217 | First |
| hCoV-19/India/MH-ACTREC-101/2020 | betacoronavirus | EPI_ISL_699757 | 23-05-2020 | India | 29 | Female | B.1.217 | First |
| hCoV-19/India/MH-ACTREC-102/2020 | betacoronavirus | EPI_ISL_699758 | 23-05-2020 | India | 22 | Female | B.1.210 | First |
| hCoV-19/India/MH-ACTREC-103/2020 | betacoronavirus | EPI_ISL_699759 | 23-05-2020 | India | 38 | Male | B.1.217 | First |
| hCoV-19/India/MH-ACTREC-104/2020 | betacoronavirus | EPI_ISL_699760 | 23-05-2020 | India | 56 | Female | B.1.1.281 | First |
| hCoV-19/India/MH-ACTREC-105/2020 | betacoronavirus | EPI_ISL_699761 | 23-05-2020 | India | 32 | Male | B.6 | First |
| hCoV-19/India/MH-ACTREC-106/2020 | betacoronavirus | EPI_ISL_699762 | 24-05-2020 | India | 42 | Female | B.1.1.281 | First |
| hCoV-19/India/MH-ACTREC-107/2020 | betacoronavirus | EPI_ISL_699763 | 24-05-2020 | India | 38 | Male | B.1.1.298 | First |
| hCoV-19/India/MH-ACTREC-108/2020 | betacoronavirus | EPI_ISL_699764 | 24-05-2020 | India | 11 | Male | B.1.217 | First |
| hCoV-19/India/MH-ACTREC-109/2020 | betacoronavirus | EPI_ISL_699765 | 24-05-2020 | India | 35 | Male | B.1.1.306 | First |
| hCoV-19/India/MH-ACTREC-110/2020 | betacoronavirus | EPI_ISL_699766 | 24-05-2020 | India | 52 | Female | B.1.1.212 | First |
| hCoV-19/India/MH-ACTREC-111/2020 | betacoronavirus | EPI_ISL_699767 | 25-05-2020 | India | 54 | Female | B.1.210 | First |
| hCoV-19/India/MH-ACTREC-112/2020 | betacoronavirus | EPI_ISL_699768 | 25-05-2020 | India | 46 | Female | B.1.210 | First |
| hCoV-19/India/MH-ACTREC-113/2020 | betacoronavirus | EPI_ISL_699769 | 25-05-2020 | India | 41 | Male | B.1.210 | First |
| hCoV-19/India/MH-ACTREC-114/2020 | betacoronavirus | EPI_ISL_699770 | 25-05-2020 | India | 2 | Female | B.1.210 | First |
| hCoV-19/India/MH-ACTREC-115/2020 | betacoronavirus | EPI_ISL_699771 | 25-05-2020 | India | 40 | Female | B.1.210 | First |
| hCoV-19/India/MH-ACTREC-116/2020 | betacoronavirus | EPI_ISL_699772 | 25-05-2020 | India | 21 | Male | B.1.1.281 | First |
| hCoV-19/India/MH-ACTREC-117/2020 | betacoronavirus | EPI_ISL_699773 | 25-05-2020 | India | 53 | Male | B.1.1.281 | First |
| hCoV-19/India/MH-ACTREC-118/2020 | betacoronavirus | EPI_ISL_699774 | 25-05-2020 | India | 48 | Female | B.1.1.281 | First |
| hCoV-19/India/MH-ACTREC-119/2020 | betacoronavirus | EPI_ISL_699775 | 25-05-2020 | India | 18 | Female | B.1.1.212 | First |
| hCoV-19/India/MH-ACTREC-120/2020 | betacoronavirus | EPI_ISL_699776 | 25-05-2020 | India | 8 | Male | B.1.210 | First |
| hCoV-19/India/MH-ACTREC-121/2020 | betacoronavirus | EPI_ISL_699777 | 25-05-2020 | India | 58 | Female | B.1.217 | First |
| hCoV-19/India/MH-ACTREC-122/2020 | betacoronavirus | EPI_ISL_699778 | 25-05-2020 | India | 57 | Male | B.1.1.298 | First |
| hCoV-19/India/MH-ACTREC-123/2020 | betacoronavirus | EPI_ISL_699779 | 25-05-2020 | India | 56 | Male | B.1.1.281 | First |
| hCoV-19/India/MH-ACTREC-124/2020 | betacoronavirus | EPI_ISL_699780 | 25-05-2020 | India | 40 | Male | B.1.1.281 | First |
| hCoV-19/India/MH-ACTREC-125/2020 | betacoronavirus | EPI_ISL_699781 | 25-05-2020 | India | 58 | Male | B.1.1.281 | First |
| hCoV-19/India/MH-ACTREC-126/2020 | betacoronavirus | EPI_ISL_699782 | 25-05-2020 | India | 39 | Male | B.1.1.281 | First |
| hCoV-19/India/MH-ACTREC-127/2020 | betacoronavirus | EPI_ISL_699783 | 25-05-2020 | India | 44 | Male | B.1.210 | First |
| hCoV-19/India/MH-ACTREC-128/2020 | betacoronavirus | EPI_ISL_699784 | 26-05-2020 | India | 45 | Female | B.1.210 | First |
| hCoV-19/India/MH-ACTREC-129/2020 | betacoronavirus | EPI_ISL_699785 | 26-05-2020 | India | 7 | Male | B.1.217 | First |
| hCoV-19/India/MH-ACTREC-130/2020 | betacoronavirus | EPI_ISL_699786 | 26-05-2020 | India | 61 | Female | B.1.217 | First |
| hCoV-19/India/MH-ACTREC-131/2020 | betacoronavirus | EPI_ISL_699787 | 26-05-2020 | India | 58 | Male | B.1.1.281 | First |
| hCoV-19/India/MH-ACTREC-132/2020 | betacoronavirus | EPI_ISL_699788 | 26-05-2020 | India | 40 | Female | B.1.210 | First |
| hCoV-19/India/MH-ACTREC-133/2020 | betacoronavirus | EPI_ISL_699789 | 27-05-2020 | India | 25 | Male | B.1.210 | First |
| hCoV-19/India/MH-ACTREC-134/2020 | betacoronavirus | EPI_ISL_699790 | 28-05-2020 | India | 29 | Male | B.1.210 | First |
| hCoV-19/India/MH-ACTREC-135/2020 | betacoronavirus | EPI_ISL_699791 | 28-05-2020 | India | 74 | Female | B.1.1.306 | First |
| hCoV-19/India/MH-ACTREC-136/2020 | betacoronavirus | EPI_ISL_699792 | 28-05-2020 | India | 38 | Female | B.1.1.306 | First |
| hCoV-19/India/MH-ACTREC-137/2020 | betacoronavirus | EPI_ISL_699793 | 28-05-2020 | India | 20 | Male | B.1.1.306 | First |
| hCoV-19/India/MH-ACTREC-138/2020 | betacoronavirus | EPI_ISL_699794 | 28-05-2020 | India | 16 | Female | B.1.1.306 | First |
| hCoV-19/India/MH-ACTREC-139/2020 | betacoronavirus | EPI_ISL_699795 | 28-05-2020 | India | 12 | Male | B.1.210 | First |
| hCoV-19/India/MH-ACTREC-140/2020 | betacoronavirus | EPI_ISL_699796 | 28-05-2020 | India | 8 | Male | B.1.210 | First |
| hCoV-19/India/MH-ACTREC-141/2020 | betacoronavirus | EPI_ISL_699797 | 28-05-2020 | India | 42 | Male | B.1.210 | First |
| hCoV-19/India/MH-ACTREC-142/2020 | betacoronavirus | EPI_ISL_699798 | 29-05-2020 | India | 60 | Male | B.1.210 | First |
| hCoV-19/India/MH-ACTREC-143/2020 | betacoronavirus | EPI_ISL_699799 | 29-05-2020 | India | 57 | Male | B.1.217 | First |
| hCoV-19/India/MH-ACTREC-144/2020 | betacoronavirus | EPI_ISL_699800 | 29-05-2020 | India | 50 | Female | B.6 | First |
| hCoV-19/India/MH-ACTREC-145/2020 | betacoronavirus | EPI_ISL_699801 | 29-05-2020 | India | 46 | Female | B.1.1.281 | First |
| hCoV-19/India/MH-ACTREC-146/2020 | betacoronavirus | EPI_ISL_699802 | 29-05-2020 | India | 31 | Male | B.1.210 | First |
| hCoV-19/India/MH-ACTREC-147/2020 | betacoronavirus | EPI_ISL_699803 | 29-05-2020 | India | 30 | Male | B.1.210 | First |
| hCoV-19/India/MH-ACTREC-148/2020 | betacoronavirus | EPI_ISL_699804 | 30-05-2020 | India | 9 | Male | B.1.1.281 | First |
| hCoV-19/India/MH-ACTREC-149/2020 | betacoronavirus | EPI_ISL_699805 | 31-05-2020 | India | 18 | Male | B.1.1.298 | First |
| hCoV-19/India/MH-ACTREC-150/2020 | betacoronavirus | EPI_ISL_699806 | 31-05-2020 | India | 52 | Male | B.1.1.298 | First |
| hCoV-19/India/MH-ACTREC-151/2020 | betacoronavirus | EPI_ISL_699807 | 02-06-2020 | India | 49 | Female | B.1.210 | First |
| hCoV-19/India/MH-ACTREC-152/2020 | betacoronavirus | EPI_ISL_699808 | 02-06-2020 | India | 50 | Male | B.1.1.281 | First |
| hCoV-19/India/MH-ACTREC-153/2020 | betacoronavirus | EPI_ISL_699809 | 02-06-2020 | India | 44 | Male | B.1.217 | First |
| hCoV-19/India/MH-ACTREC-154/2020 | betacoronavirus | EPI_ISL_699810 | 02-06-2020 | India | 43 | Female | B.1.1.281 | First |
| hCoV-19/India/MH-ACTREC-155/2020 | betacoronavirus | EPI_ISL_699811 | 02-06-2020 | India | 53 | Male | B.1.1.212 | First |
| hCoV-19/India/MH-ACTREC-156/2020 | betacoronavirus | EPI_ISL_699812 | 04-06-2020 | India | 33 | Male | B.1.1.281 | First |
| hCoV-19/India/MH-ACTREC-157/2020 | betacoronavirus | EPI_ISL_699813 | 04-06-2020 | India | 33 | Female | B.1.210 | First |
| hCoV-19/India/MH-ACTREC-158/2020 | betacoronavirus | EPI_ISL_699814 | 04-06-2020 | India | 34 | Male | B.1.210 | First |
| hCoV-19/India/MH-ACTREC-159/2020 | betacoronavirus | EPI_ISL_699815 | 04-06-2020 | India | 50 | Female | B.1.1.281 | First |
| hCoV-19/India/MH-ACTREC-160/2020 | betacoronavirus | EPI_ISL_699816 | 04-06-2020 | India | 11 | Male | B.1.1.281 | First |
| hCoV-19/India/MH-ACTREC-161/2020 | betacoronavirus | EPI_ISL_699817 | 04-06-2020 | India | 51 | Male | B.1.1.212 | First |
| hCoV-19/India/MH-ACTREC-162/2020 | betacoronavirus | EPI_ISL_699818 | 04-06-2020 | India | 35 | Male | B.1.210 | First |
| hCoV-19/India/MH-ACTREC-163/2020 | betacoronavirus | EPI_ISL_699819 | 04-06-2020 | India | 40 | Male | B.1.210 | First |
| hCoV-19/India/MH-ACTREC-164/2020 | betacoronavirus | EPI_ISL_699820 | 04-06-2020 | India | 50 | Male | B.1.1.281 | First |
| hCoV-19/India/MH-ACTREC-165/2020 | betacoronavirus | EPI_ISL_699821 | 04-06-2020 | India | 23 | Female | B.1.1.281 | First |
| hCoV-19/India/MH-ACTREC-166/2020 | betacoronavirus | EPI_ISL_699822 | 05-06-2020 | India | 38 | Male | B.1.1.281 | First |
| hCoV-19/India/MH-ACTREC-167/2020 | betacoronavirus | EPI_ISL_699823 | 06-06-2020 | India | 44 | Male | B.1.247 | First |
| hCoV-19/India/MH-ACTREC-168/2020 | betacoronavirus | EPI_ISL_699824 | 06-06-2020 | India | 46 | Female | B.1.210 | First |
| hCoV-19/India/MH-ACTREC-169/2020 | betacoronavirus | EPI_ISL_723046 | 08-06-2020 | India | 30 | Female | B.1.210 | First |
| hCoV-19/India/MH-ACTREC-170/2020 | betacoronavirus | EPI_ISL_699825 | 11-06-2020 | India | 22 | Female | B.1.1 | First |
| hCoV-19/India/MH-ACTREC-171/2020 | betacoronavirus | EPI_ISL_699826 | 11-06-2020 | India | 46 | Male | B.1.1.306 | First |
| hCoV-19/India/MH-ACTREC-172/2020 | betacoronavirus | EPI_ISL_699827 | 11-06-2020 | India | 24 | Female | B.1.5 | First |
| hCoV-19/India/MH-ACTREC-173/2020 | betacoronavirus | EPI_ISL_699828 | 12-06-2020 | India | 36 | Male | B.1.1.281 | First |
| hCoV-19/India/MH-ACTREC-174/2020 | betacoronavirus | EPI_ISL_699829 | 13-06-2020 | India | 45 | Male | B.1.247 | First |
| hCoV-19/India/MH-ACTREC-175/2020 | betacoronavirus | EPI_ISL_699830 | 15-06-2020 | India | 38 | Male | B.1.210 | First |
| hCoV-19/India/MH-ACTREC-176/2020 | betacoronavirus | EPI_ISL_699831 | 15-06-2020 | India | 50 | Female | B.1.1.281 | First |
| hCoV-19/India/MH-ACTREC-177/2020 | betacoronavirus | EPI_ISL_699832 | 15-06-2020 | India | 57 | Male | B.1.1.281 | First |
| hCoV-19/India/MH-ACTREC-178/2020 | betacoronavirus | EPI_ISL_699833 | 15-06-2020 | India | 54 | Female | B.1.1.281 | First |
| hCoV-19/India/MH-ACTREC-179/2020 | betacoronavirus | EPI_ISL_699834 | 15-06-2020 | India | 19 | Female | B.1.1.281 | First |
| hCoV-19/India/MH-ACTREC-180/2020 | betacoronavirus | EPI_ISL_699835 | 15-06-2020 | India | 65 | Male | B.1.217 | First |
| hCoV-19/India/MH-ACTREC-181/2020 | betacoronavirus | EPI_ISL_699836 | 17-06-2020 | India | 36 | Female | B.1.1.281 | First |
| hCoV-19/India/MH-ACTREC-182/2020 | betacoronavirus | EPI_ISL_699837 | 17-06-2020 | India | 42 | Male | B.1.1.281 | First |
| hCoV-19/India/MH-ACTREC-183/2020 | betacoronavirus | EPI_ISL_699838 | 17-06-2020 | India | 34 | Female | B.1.1.281 | First |
| hCoV-19/India/MH-ACTREC-184/2020 | betacoronavirus | EPI_ISL_699839 | 17-06-2020 | India | 34 | Male | B.1.1.281 | First |
| hCoV-19/India/MH-ACTREC-185/2020 | betacoronavirus | EPI_ISL_699840 | 18-06-2020 | India | 31 | Female | B.1.1.281 | First |
| hCoV-19/India/MH-ACTREC-186/2020 | betacoronavirus | EPI_ISL_699841 | 19-06-2020 | India | 65 | Female | B.1.1.281 | First |
| hCoV-19/India/MH-ACTREC-187/2020 | betacoronavirus | EPI_ISL_723047 | 19-06-2020 | India | 28 | Female | B.1.1 | First |
| hCoV-19/India/MH-ACTREC-188/2020 | betacoronavirus | EPI_ISL_699842 | 22-06-2020 | India | 43 | Male | B.1.1 | First |
| hCoV-19/India/MH-ACTREC-189/2020 | betacoronavirus | EPI_ISL_699843 | 22-06-2020 | India | 15 | Male | B.1.1.281 | First |
| hCoV-19/India/MH-ACTREC-190/2020 | betacoronavirus | EPI_ISL_699844 | 22-06-2020 | India | 9 | Male | B.1.1.281 | First |
| hCoV-19/India/MH-ACTREC-191/2020 | betacoronavirus | EPI_ISL_699845 | 22-06-2020 | India | 62 | Male | B.1.1.281 | First |
| hCoV-19/India/MH-ACTREC-192/2020 | betacoronavirus | EPI_ISL_699846 | 22-06-2020 | India | 23 | Female | B.1.1.281 | First |
| hCoV-19/India/MH-ACTREC-193/2020 | betacoronavirus | EPI_ISL_699847 | 22-06-2020 | India | 19 | Male | B.1.1.281 | First |
| hCoV-19/India/MH-ACTREC-194/2020 | betacoronavirus | EPI_ISL_699848 | 22-06-2020 | India | 24 | Male | B.1.36.1 | First |
| hCoV-19/India/MH-ACTREC-195/2020 | betacoronavirus | EPI_ISL_699849 | 22-06-2020 | India | 56 | Male | B.1.36.1 | First |
| hCoV-19/India/MH-ACTREC-196/2020 | betacoronavirus | EPI_ISL_699850 | 22-06-2020 | India | 51 | Female | B.1.36.1 | First |
| hCoV-19/India/MH-ACTREC-197/2020 | betacoronavirus | EPI_ISL_699851 | 22-06-2020 | India | 69 | Male | B.1.1.306 | First |
| hCoV-19/India/MH-ACTREC-198/2020 | betacoronavirus | EPI_ISL_699852 | 23-06-2020 | India | 36 | Male | B.1.1.281 | First |
| hCoV-19/India/MH-ACTREC-199/2020 | betacoronavirus | EPI_ISL_699853 | 23-06-2020 | India | 48 | Male | B.1.1.281 | First |
| hCoV-19/India/MH-ACTREC-200/2020 | betacoronavirus | EPI_ISL_699854 | 25-06-2020 | India | 33 | Male | B.1.1 | First |
| hCoV-19/India/MH-ACTREC-201/2020 | betacoronavirus | EPI_ISL_699855 | 25-06-2020 | India | 39 | Male | B.1.210 | First |
| hCoV-19/India/MH-ACTREC-202/2020 | betacoronavirus | EPI_ISL_699856 | 25-06-2020 | India | 17 | Female | B.1.1.281 | First |
| hCoV-19/India/MH-ACTREC-203/2020 | betacoronavirus | EPI_ISL_699857 | 25-06-2020 | India | 52 | Male | B.1.1.281 | First |
| hCoV-19/India/MH-ACTREC-204/2020 | betacoronavirus | EPI_ISL_699858 | 25-06-2020 | India | 43 | Male | B.1.210 | First |
| hCoV-19/India/MH-ACTREC-205/2020 | betacoronavirus | EPI_ISL_699859 | 26-06-2020 | India | 62 | Female | B.1.1.281 | First |
| hCoV-19/India/MH-ACTREC-206/2020 | betacoronavirus | EPI_ISL_699860 | 26-06-2020 | India | 37 | Female | B.1.210 | First |
| hCoV-19/India/MH-ACTREC-207/2020 | betacoronavirus | EPI_ISL_699861 | 26-06-2020 | India | 13 | Female | B.1.210 | First |
| hCoV-19/India/MH-ACTREC-208/2020 | betacoronavirus | EPI_ISL_699862 | 26-06-2020 | India | 7 | Male | B.1.210 | First |
| hCoV-19/India/MH-ACTREC-209/2020 | betacoronavirus | EPI_ISL_699863 | 27-06-2020 | India | 45 | Male | B.1.210 | First |
| hCoV-19/India/MH-ACTREC-210/2020 | betacoronavirus | EPI_ISL_699864 | 27-06-2020 | India | 30 | Female | B.1.210 | First |
| hCoV-19/India/MH-ACTREC-211/2020 | betacoronavirus | EPI_ISL_699865 | 27-06-2020 | India | 30 | Male | B.1.210 | First |
| hCoV-19/India/MH-ACTREC-212/2020 | betacoronavirus | EPI_ISL_699866 | 29-06-2020 | India | 67 | Female | B.1.1.212 | First |
| hCoV-19/India/MH-ACTREC-213/2020 | betacoronavirus | EPI_ISL_699867 | 29-06-2020 | India | 42 | Male | B.1.217 | First |
| hCoV-19/India/MH-ACTREC-214/2020 | betacoronavirus | EPI_ISL_699868 | 29-06-2020 | India | 36 | Male | B.1.1 | First |
| hCoV-19/India/MH-ACTREC-215/2020 | betacoronavirus | EPI_ISL_699869 | 29-06-2020 | India | 27 | Male | B.1.1 | First |
| hCoV-19/India/MH-ACTREC-216/2020 | betacoronavirus | EPI_ISL_699870 | 29-06-2020 | India | 45 | Male | B.1.1.281 | First |
| hCoV-19/India/MH-ACTREC-217/2020 | betacoronavirus | EPI_ISL_699871 | 29-06-2020 | India | 53 | Male | B.1.1.281 | First |
| hCoV-19/India/MH-ACTREC-218/2020 | betacoronavirus | EPI_ISL_699872 | 29-06-2020 | India | 36 | Male | B.1.210 | First |
| hCoV-19/India/MH-ACTREC-219/2020 | betacoronavirus | EPI_ISL_699873 | 29-06-2020 | India | 23 | Male | B.1.1.281 | First |
| hCoV-19/India/MH-ACTREC-220/2020 | betacoronavirus | EPI_ISL_699874 | 30-06-2020 | India | 47 | Female | B.1.1.281 | First |
| hCoV-19/India/MH-ACTREC-221/2020 | betacoronavirus | EPI_ISL_699875 | 30-06-2020 | India | 45 | Male | B.1.210 | First |
| hCoV-19/India/MH-ACTREC-222/2020 | betacoronavirus | EPI_ISL_699876 | 30-06-2020 | India | 53 | Male | B.1.217 | First |
| hCoV-19/India/MH-ACTREC-223/2020 | betacoronavirus | EPI_ISL_699877 | 30-06-2020 | India | 42 | Female | B.1.1.281 | First |
| hCoV-19/India/MH-ACTREC-224/2020 | betacoronavirus | EPI_ISL_699878 | 01-07-2020 | India | 42 | Female | B.1.217 | First |
| hCoV-19/India/MH-ACTREC-225/2020 | betacoronavirus | EPI_ISL_699879 | 01-07-2020 | India | 29 | Female | B.1.1.281 | First |
| hCoV-19/India/MH-ACTREC-226/2020 | betacoronavirus | EPI_ISL_699880 | 01-07-2020 | India | 32 | Female | B.1.210 | First |
| hCoV-19/India/MH-ACTREC-227/2020 | betacoronavirus | EPI_ISL_699881 | 02-07-2020 | India | 28 | Male | B.1.1.281 | First |
| hCoV-19/India/MH-ACTREC-228/2020 | betacoronavirus | EPI_ISL_699882 | 02-07-2020 | India | 48 | Male | B.1.210 | First |
| hCoV-19/India/MH-ACTREC-229/2020 | betacoronavirus | EPI_ISL_723048 | 02-07-2020 | India | 38 | Male | B.1.1.281 | First |
| hCoV-19/India/MH-ACTREC-230/2020 | betacoronavirus | EPI_ISL_699883 | 02-07-2020 | India | 35 | Male | B.1.210 | First |
| hCoV-19/India/MH-ACTREC-231/2020 | betacoronavirus | EPI_ISL_699884 | 02-07-2020 | India | 32 | Male | B.1.1.306 | First |
| hCoV-19/India/MH-ACTREC-232/2020 | betacoronavirus | EPI_ISL_699885 | 03-07-2020 | India | 33 | Male | B.1.210 | First |
| hCoV-19/India/MH-ACTREC-233/2020 | betacoronavirus | EPI_ISL_699886 | 03-07-2020 | India | 29 | Male | B.1.1.306 | First |
| hCoV-19/India/MH-ACTREC-234/2020 | betacoronavirus | EPI_ISL_699887 | 04-07-2020 | India | 52 | Female | B.1.1.306 | First |
| hCoV-19/India/MH-ACTREC-235/2020 | betacoronavirus | EPI_ISL_699888 | 04-07-2020 | India | 42 | Female | B.1.1.281 | First |
| hCoV-19/India/MH-ACTREC-236/2020 | betacoronavirus | EPI_ISL_699889 | 05-07-2020 | India | 50 | Male | B.1.210 | First |
| hCoV-19/India/MH-ACTREC-237/2020 | betacoronavirus | EPI_ISL_699890 | 06-07-2020 | India | 46 | Male | B.1.1 | First |
| hCoV-19/India/MH-ACTREC-238/2020 | betacoronavirus | EPI_ISL_699891 | 06-07-2020 | India | 55 | Male | B.1.1.281 | First |
| hCoV-19/India/MH-ACTREC-239/2020 | betacoronavirus | EPI_ISL_699892 | 06-07-2020 | India | 62 | Male | B.1.210 | First |
| hCoV-19/India/MH-ACTREC-240/2020 | betacoronavirus | EPI_ISL_699893 | 06-07-2020 | India | 18 | Male | B.1.217 | First |
| hCoV-19/India/MH-ACTREC-241/2020 | betacoronavirus | EPI_ISL_699894 | 06-07-2020 | India | 59 | Male | B.1.1.281 | First |
| hCoV-19/India/MH-ACTREC-242/2020 | betacoronavirus | EPI_ISL_699895 | 06-07-2020 | India | 16 | Male | B.1.1.281 | First |
| hCoV-19/India/MH-ACTREC-243/2020 | betacoronavirus | EPI_ISL_699896 | 06-07-2020 | India | 50 | Male | B.1.5 | First |
| hCoV-19/India/MH-ACTREC-244/2020 | betacoronavirus | EPI_ISL_699897 | 06-07-2020 | India | 37 | Male | B.1.1.212 | First |
| hCoV-19/India/MH-ACTREC-245/2020 | betacoronavirus | EPI_ISL_699898 | 07-07-2020 | India | 25 | Male | B.1.1.306 | First |
| hCoV-19/India/MH-ACTREC-246/2020 | betacoronavirus | EPI_ISL_699899 | 07-07-2020 | India | 34 | Male | B.1 | First |
| hCoV-19/India/MH-ACTREC-247/2020 | betacoronavirus | EPI_ISL_699900 | 07-07-2020 | India | 29 | Female | B.1.1.306 | First |
| hCoV-19/India/MH-ACTREC-248/2020 | betacoronavirus | EPI_ISL_723049 | 07-07-2020 | India | 62 | Female | B.1.1.212 | First |
| hCoV-19/India/MH-ACTREC-249/2020 | betacoronavirus | EPI_ISL_699901 | 07-07-2020 | India | 44 | Male | B.1.1.281 | First |
| hCoV-19/India/MH-ACTREC-250/2020 | betacoronavirus | EPI_ISL_699902 | 07-07-2020 | India | 41 | Male | B.1.1.281 | First |
| hCoV-19/India/MH-ACTREC-251/2020 | betacoronavirus | EPI_ISL_699903 | 07-07-2020 | India | 50 | Male | B.1.1.281 | First |
| hCoV-19/India/MH-ACTREC-252/2020 | betacoronavirus | EPI_ISL_699904 | 07-07-2020 | India | 28 | Female | B.1.210 | First |
| hCoV-19/India/MH-ACTREC-253/2020 | betacoronavirus | EPI_ISL_699905 | 08-07-2020 | India | 29 | Male | B.1.1.306 | First |
| hCoV-19/India/MH-ACTREC-254/2020 | betacoronavirus | EPI_ISL_699906 | 08-07-2020 | India | 32 | Male | B.1.1.281 | First |
| hCoV-19/India/MH-ACTREC-255/2020 | betacoronavirus | EPI_ISL_699907 | 08-07-2020 | India | 34 | Male | B.1.1.281 | First |
| hCoV-19/India/MH-ACTREC-256/2020 | betacoronavirus | EPI_ISL_699908 | 08-07-2020 | India | 55 | Male | B.1.1.281 | First |
| hCoV-19/India/MH-ACTREC-257/2020 | betacoronavirus | EPI_ISL_699909 | 08-07-2020 | India | 28 | Male | B.1.1.281 | First |
| hCoV-19/India/MH-ACTREC-258/2020 | betacoronavirus | EPI_ISL_699910 | 08-07-2020 | India | 55 | Male | B.1.1.281 | First |
| hCoV-19/India/MH-ACTREC-259/2020 | betacoronavirus | EPI_ISL_699911 | 08-07-2020 | India | 54 | Female | B.1.1.281 | First |
| hCoV-19/India/MH-ACTREC-260/2020 | betacoronavirus | EPI_ISL_699912 | 08-07-2020 | India | 24 | Male | B.1.1.281 | First |
| hCoV-19/India/MH-ACTREC-261/2020 | betacoronavirus | EPI_ISL_699913 | 08-07-2020 | India | 45 | Female | B.1.1.281 | First |
| hCoV-19/India/MH-ACTREC-262/2020 | betacoronavirus | EPI_ISL_699914 | 08-07-2020 | India | 28 | Male | B.1.1.281 | First |
| hCoV-19/India/MH-ACTREC-263/2020 | betacoronavirus | EPI_ISL_699915 | 08-07-2020 | India | 36 | Female | B.1.1.281 | First |
| hCoV-19/India/MH-ACTREC-264/2020 | betacoronavirus | EPI_ISL_699916 | 08-07-2020 | India | 12 | Male | B.1.1.281 | First |
| hCoV-19/India/MH-ACTREC-265/2020 | betacoronavirus | EPI_ISL_699917 | 08-07-2020 | India | 28 | Male | B.1.1.306 | First |
| hCoV-19/India/MH-ACTREC-266/2020 | betacoronavirus | EPI_ISL_699918 | 08-07-2020 | India | 58 | Male | B.1.1.281 | First |
| hCoV-19/India/MH-ACTREC-267/2020 | betacoronavirus | EPI_ISL_699919 | 09-07-2020 | India | 38 | Male | B.1.1.179 | First |
| hCoV-19/India/MH-ACTREC-268/2020 | betacoronavirus | EPI_ISL_699920 | 09-07-2020 | India | 37 | Male | B.1.1.281 | First |
| hCoV-19/India/MH-ACTREC-269/2020 | betacoronavirus | EPI_ISL_699921 | 09-07-2020 | India | 42 | Female | B.1.1.281 | First |
| hCoV-19/India/MH-ACTREC-270/2020 | betacoronavirus | EPI_ISL_699922 | 09-07-2020 | India | 33 | Male | B.1.1.281 | First |
| hCoV-19/India/MH-ACTREC-271/2020 | betacoronavirus | EPI_ISL_699923 | 10-07-2020 | India | 22 | Male | B.1.1.281 | First |
| hCoV-19/India/MH-ACTREC-272/2020 | betacoronavirus | EPI_ISL_699924 | 10-07-2020 | India | 34 | Female | B.1.1.281 | First |
| hCoV-19/India/MH-ACTREC-273/2020 | betacoronavirus | EPI_ISL_699925 | 10-07-2020 | India | 31 | Male | B.1.217 | First |
| hCoV-19/India/MH-ACTREC-274/2020 | betacoronavirus | EPI_ISL_699926 | 10-07-2020 | India | 77 | Male | B.1.1.281 | First |
| hCoV-19/India/MH-ACTREC-275/2020 | betacoronavirus | EPI_ISL_699927 | 10-07-2020 | India | 48 | Female | B.1.1.281 | First |
| hCoV-19/India/MH-ACTREC-276/2020 | betacoronavirus | EPI_ISL_699928 | 10-07-2020 | India | 60 | Female | B.1.1.281 | First |
| hCoV-19/India/MH-ACTREC-277/2020 | betacoronavirus | EPI_ISL_699929 | 10-07-2020 | India | 35 | Male | B.1.1.281 | First |
| hCoV-19/India/MH-ACTREC-278/2020 | betacoronavirus | EPI_ISL_699930 | 11-07-2020 | India | 55 | Female | B.1.1.281 | First |
| hCoV-19/India/MH-ACTREC-279/2020 | betacoronavirus | EPI_ISL_699931 | 11-07-2020 | India | 27 | Female | B.1.1.281 | First |
| hCoV-19/India/MH-ACTREC-280/2020 | betacoronavirus | EPI_ISL_699932 | 11-07-2020 | India | 38 | Male | B.1.210 | First |
| hCoV-19/India/MH-ACTREC-281/2020 | betacoronavirus | EPI_ISL_699933 | 11-07-2020 | India | 45 | Male | B.1.210 | First |
| hCoV-19/India/MH-ACTREC-282/2020 | betacoronavirus | EPI_ISL_699934 | 11-07-2020 | India | 38 | Male | B.1.210 | First |
| hCoV-19/India/MH-ACTREC-283/2020 | betacoronavirus | EPI_ISL_699935 | 11-07-2020 | India | 54 | Male | B.1.1.281 | First |
| hCoV-19/India/MH-ACTREC-284/2020 | betacoronavirus | EPI_ISL_699936 | 12-07-2020 | India | 58 | Female | B.1.210 | First |
| hCoV-19/India/MH-ACTREC-285/2020 | betacoronavirus | EPI_ISL_699937 | 12-07-2020 | India | 60 | Male | B.1.217 | First |
| hCoV-19/India/MH-ACTREC-286/2020 | betacoronavirus | EPI_ISL_699938 | 13-07-2020 | India | 42 | Female | B.1.1.281 | First |
| hCoV-19/India/MH-ACTREC-287/2020 | betacoronavirus | EPI_ISL_699939 | 13-07-2020 | India | 33 | Male | B.1.210 | First |
| hCoV-19/India/MH-ACTREC-288/2020 | betacoronavirus | EPI_ISL_699940 | 13-07-2020 | India | 30 | Male | B.1.1.281 | First |
| hCoV-19/India/MH-ACTREC-289/2020 | betacoronavirus | EPI_ISL_699941 | 13-07-2020 | India | 59 | Male | B.1.217 | First |
| hCoV-19/India/MH-ACTREC-290/2020 | betacoronavirus | EPI_ISL_699942 | 13-07-2020 | India | 39 | Male | B.1.1.281 | First |
| hCoV-19/India/MH-ACTREC-291/2020 | betacoronavirus | EPI_ISL_699943 | 14-07-2020 | India | 53 | Female | B.1.210 | First |
| hCoV-19/India/MH-ACTREC-292/2020 | betacoronavirus | EPI_ISL_699944 | 14-07-2020 | India | 34 | Male | B.1.1.306 | First |
| hCoV-19/India/MH-ACTREC-293/2020 | betacoronavirus | EPI_ISL_699945 | 14-07-2020 | India | 38 | Female | B.1.1.306 | First |
| hCoV-19/India/MH-ACTREC-294/2020 | betacoronavirus | EPI_ISL_699946 | 14-07-2020 | India | 85 | Male | B.1.1 | First |
| hCoV-19/India/MH-ACTREC-295/2020 | betacoronavirus | EPI_ISL_699947 | 14-07-2020 | India | 16 | Female | B.1.1.64 | First |
| hCoV-19/India/MH-ACTREC-296/2020 | betacoronavirus | EPI_ISL_699948 | 14-07-2020 | India | 50 | Male | B.1.1.64 | First |
| hCoV-19/India/MH-ACTREC-297/2020 | betacoronavirus | EPI_ISL_699949 | 14-07-2020 | India | 69 | Male | B.1.1.306 | First |
| hCoV-19/India/MH-ACTREC-298/2020 | betacoronavirus | EPI_ISL_699950 | 14-07-2020 | India | 65 | Female | B.1.1.281 | First |
| hCoV-19/India/MH-ACTREC-299/2020 | betacoronavirus | EPI_ISL_699951 | 14-07-2020 | India | 56 | Male | B.1.1.281 | First |
| hCoV-19/India/MH-ACTREC-300/2020 | betacoronavirus | EPI_ISL_723050 | 14-07-2020 | India | 30 | Male | B.1.1.212 | First |
| hCoV-19/India/MH-ACTREC-301/2020 | betacoronavirus | EPI_ISL_699952 | 14-07-2020 | India | 18 | Male | B.1.1.281 | First |
| hCoV-19/India/MH-ACTREC-302/2020 | betacoronavirus | EPI_ISL_699953 | 15-07-2020 | India | 65 | Female | B.1.1.306 | First |
| hCoV-19/India/MH-ACTREC-303/2020 | betacoronavirus | EPI_ISL_699954 | 15-07-2020 | India | 27 | Male | B.1.1.281 | First |
| hCoV-19/India/MH-ACTREC-304/2020 | betacoronavirus | EPI_ISL_699955 | 15-07-2020 | India | 56 | Male | B.1.210 | First |
| hCoV-19/India/MH-ACTREC-305/2020 | betacoronavirus | EPI_ISL_699956 | 15-07-2020 | India | 26 | Male | B.1.1.306 | First |
| hCoV-19/India/MH-ACTREC-306/2020 | betacoronavirus | EPI_ISL_699957 | 15-07-2020 | India | 48 | Female | B.1.1.281 | First |
| hCoV-19/India/MH-ACTREC-307/2020 | betacoronavirus | EPI_ISL_699958 | 15-07-2020 | India | 33 | Female | B.1.1.281 | First |
| hCoV-19/India/MH-ACTREC-308/2020 | betacoronavirus | EPI_ISL_699959 | 15-07-2020 | India | 3 | Female | B.1.1.281 | First |
| hCoV-19/India/MH-ACTREC-309/2020 | betacoronavirus | EPI_ISL_699960 | 15-07-2020 | India | 39 | Female | B.1.1.281 | First |
| hCoV-19/India/MH-ACTREC-310/2020 | betacoronavirus | EPI_ISL_699961 | 15-07-2020 | India | 15 | Male | B.1.1.281 | First |
| hCoV-19/India/MH-ACTREC-311/2020 | betacoronavirus | EPI_ISL_723051 | 15-07-2020 | India | 34 | Male | B.1.1.281 | First |
| hCoV-19/India/MH-ACTREC-312/2020 | betacoronavirus | EPI_ISL_723052 | 15-07-2020 | India | 32 | Female | B.1.1.212 | First |
| hCoV-19/India/MH-ACTREC-313/2020 | betacoronavirus | EPI_ISL_699962 | 15-07-2020 | India | 66 | Male | B.1.1.281 | First |
| hCoV-19/India/MH-ACTREC-314/2020 | betacoronavirus | EPI_ISL_699963 | 16-07-2020 | India | 54 | Male | B.1.1.281 | First |
| hCoV-19/India/MH-ACTREC-315/2020 | betacoronavirus | EPI_ISL_699964 | 16-07-2020 | India | 34 | Male | B.1.1.281 | First |
| hCoV-19/India/MH-ACTREC-316/2020 | betacoronavirus | EPI_ISL_699965 | 16-07-2020 | India | 31 | Male | B.1.1.281 | First |
| hCoV-19/India/MH-ACTREC-317/2020 | betacoronavirus | EPI_ISL_699966 | 16-07-2020 | India | 61 | Female | B.1.1.281 | First |
| hCoV-19/India/MH-ACTREC-318/2020 | betacoronavirus | EPI_ISL_699967 | 16-07-2020 | India | 52 | Female | B.1.1.281 | First |
| hCoV-19/India/MH-ACTREC-319/2020 | betacoronavirus | EPI_ISL_699968 | 16-07-2020 | India | 52 | Male | B.1.217 | First |
| hCoV-19/India/MH-ACTREC-320/2020 | betacoronavirus | EPI_ISL_699969 | 16-07-2020 | India | 60 | Male | B.1.1.281 | First |
| hCoV-19/India/MH-ACTREC-321/2020 | betacoronavirus | EPI_ISL_699970 | 16-07-2020 | India | 66 | Female | B.1.1.281 | First |
| hCoV-19/India/MH-ACTREC-322/2020 | betacoronavirus | EPI_ISL_699971 | 17-07-2020 | India | 44 | Male | B.1.210 | First |
| hCoV-19/India/MH-ACTREC-323/2020 | betacoronavirus | EPI_ISL_699972 | 17-07-2020 | India | 18 | Female | B.1.210 | First |
| hCoV-19/India/MH-ACTREC-324/2020 | betacoronavirus | EPI_ISL_699973 | 17-07-2020 | India | 52 | Male | B.1.1.281 | First |
| hCoV-19/India/MH-ACTREC-325/2020 | betacoronavirus | EPI_ISL_699974 | 17-07-2020 | India | 39 | Male | B.1.1.281 | First |
| hCoV-19/India/MH-ACTREC-326/2020 | betacoronavirus | EPI_ISL_699975 | 17-07-2020 | India | 34 | Male | B.1.1.281 | First |
| hCoV-19/India/MH-ACTREC-327/2020 | betacoronavirus | EPI_ISL_699976 | 17-07-2020 | India | 38 | Male | B.1.1.281 | First |
| hCoV-19/India/MH-ACTREC-328/2020 | betacoronavirus | EPI_ISL_699977 | 18-07-2020 | India | 36 | Female | B.1.1.281 | First |
| hCoV-19/India/MH-ACTREC-329/2020 | betacoronavirus | EPI_ISL_699978 | 18-07-2020 | India | 43 | Male | B.1.1.281 | First |
| hCoV-19/India/MH-ACTREC-330/2020 | betacoronavirus | EPI_ISL_699979 | 19-07-2020 | India | 34 | Male | B.1.1.281 | First |
| hCoV-19/India/MH-ACTREC-331/2020 | betacoronavirus | EPI_ISL_699980 | 19-07-2020 | India | 48 | Female | B.1.1.281 | First |
| hCoV-19/India/MH-ACTREC-332/2020 | betacoronavirus | EPI_ISL_699981 | 19-07-2020 | India | 50 | Female | B.1.1.281 | First |
| hCoV-19/India/MH-ACTREC-333/2020 | betacoronavirus | EPI_ISL_699982 | 19-07-2020 | India | 24 | Female | B.1.1.281 | First |
| hCoV-19/India/MH-ACTREC-334/2020 | betacoronavirus | EPI_ISL_699983 | 19-07-2020 | India | 37 | Male | B.1.1.281 | First |
| hCoV-19/India/MH-ACTREC-335/2020 | betacoronavirus | EPI_ISL_699984 | 19-07-2020 | India | 74 | Male | B.1.1.281 | First |
| hCoV-19/India/MH-ACTREC-336/2020 | betacoronavirus | EPI_ISL_699985 | 19-07-2020 | India | 54 | Female | B.1.1.281 | First |
| hCoV-19/India/MH-ACTREC-337/2020 | betacoronavirus | EPI_ISL_699986 | 19-07-2020 | India | 33 | Male | B.1.1.281 | First |
| hCoV-19/India/MH-ACTREC-338/2020 | betacoronavirus | EPI_ISL_699987 | 20-07-2020 | India | 29 | Male | B.1.1.281 | First |
| hCoV-19/India/MH-ACTREC-339/2020 | betacoronavirus | EPI_ISL_699988 | 20-07-2020 | India | 49 | Male | B.1.1.281 | First |
| hCoV-19/India/MH-ACTREC-340/2020 | betacoronavirus | EPI_ISL_699989 | 20-07-2020 | India | 33 | Male | B.1.210 | First |
| hCoV-19/India/MH-ACTREC-341/2020 | betacoronavirus | EPI_ISL_699990 | 20-07-2020 | India | 35 | Male | B.1.1.281 | First |
| hCoV-19/India/MH-ACTREC-342/2020 | betacoronavirus | EPI_ISL_699991 | 20-07-2020 | India | 62 | Male | B.1.1.281 | First |
| hCoV-19/India/MH-ACTREC-343/2020 | betacoronavirus | EPI_ISL_699992 | 20-07-2020 | India | 22 | Male | B.1.1.212 | First |
| hCoV-19/India/MH-ACTREC-344/2020 | betacoronavirus | EPI_ISL_699993 | 21-07-2020 | India | 42 | Male | B.1.5 | First |
| hCoV-19/India/MH-ACTREC-345/2020 | betacoronavirus | EPI_ISL_699994 | 22-07-2020 | India | 26 | Male | B.1.1.281 | First |
| hCoV-19/India/MH-ACTREC-346/2020 | betacoronavirus | EPI_ISL_699995 | 22-07-2020 | India | 57 | Female | B.1.210 | First |
| hCoV-19/India/MH-ACTREC-347/2020 | betacoronavirus | EPI_ISL_699996 | 22-07-2020 | India | 89 | Female | B.1.1.281 | First |
| hCoV-19/India/MH-ACTREC-348/2020 | betacoronavirus | EPI_ISL_699997 | 22-07-2020 | India | 45 | Male | B.1.1 | First |
| hCoV-19/India/MH-ACTREC-349/2020 | betacoronavirus | EPI_ISL_699998 | 22-07-2020 | India | 24 | Female | B.1.1.281 | First |
| hCoV-19/India/MH-ACTREC-350/2020 | betacoronavirus | EPI_ISL_699999 | 22-07-2020 | India | 25 | Male | B.1.210 | First |
| hCoV-19/India/MH-ACTREC-351/2020 | betacoronavirus | EPI_ISL_700000 | 22-07-2020 | India | 37 | Male | B.1.1.281 | First |
| hCoV-19/India/MP-ACTREC-352/2020 | betacoronavirus | EPI_ISL_700001 | 22-07-2020 | India | 60 | Male | B.1.1.281 | First |
| hCoV-19/India/MH-ACTREC-353/2020 | betacoronavirus | EPI_ISL_700002 | 22-07-2020 | India | 45 | Male | B.1.1.281 | First |
| hCoV-19/India/MH-ACTREC-354/2020 | betacoronavirus | EPI_ISL_700003 | 22-07-2020 | India | 17 | Male | B.1.1.281 | First |
| hCoV-19/India/MH-ACTREC-355/2020 | betacoronavirus | EPI_ISL_700004 | 22-07-2020 | India | 63 | Male | B.1.1.281 | First |
| hCoV-19/India/MH-ACTREC-356/2020 | betacoronavirus | EPI_ISL_700005 | 23-07-2020 | India | 46 | Male | B.1.1.281 | First |
| hCoV-19/India/MH-ACTREC-357/2020 | betacoronavirus | EPI_ISL_700006 | 23-07-2020 | India | 29 | Male | B.1.210 | First |
| hCoV-19/India/MH-ACTREC-358/2020 | betacoronavirus | EPI_ISL_700007 | 23-07-2020 | India | 56 | Female | B.1.210 | First |
| hCoV-19/India/MH-ACTREC-359/2020 | betacoronavirus | EPI_ISL_700008 | 23-07-2020 | India | 32 | Male | B.1.217 | First |
| hCoV-19/India/MH-ACTREC-360/2020 | betacoronavirus | EPI_ISL_700009 | 23-07-2020 | India | 42 | Female | B.1.1.281 | First |
| hCoV-19/India/MH-ACTREC-361/2020 | betacoronavirus | EPI_ISL_700010 | 23-07-2020 | India | 50 | Female | B.1.1.281 | First |
| hCoV-19/India/MH-ACTREC-362/2020 | betacoronavirus | EPI_ISL_700011 | 23-07-2020 | India | 54 | Male | B.1.1.281 | First |
| hCoV-19/India/MH-ACTREC-363/2020 | betacoronavirus | EPI_ISL_700012 | 23-07-2020 | India | 65 | Male | B.1.1.281 | First |
| hCoV-19/India/MH-ACTREC-364/2020 | betacoronavirus | EPI_ISL_700013 | 23-07-2020 | India | 50 | Female | B.1.1.281 | First |
| hCoV-19/India/MH-ACTREC-365/2020 | betacoronavirus | EPI_ISL_700014 | 24-07-2020 | India | 16 | Female | B.1.1.306 | First |
| hCoV-19/India/MH-ACTREC-366/2020 | betacoronavirus | EPI_ISL_700015 | 24-07-2020 | India | 31 | Female | B.1.1 | First |
| hCoV-19/India/MH-ACTREC-367/2020 | betacoronavirus | EPI_ISL_700016 | 24-07-2020 | India | 25 | Male | B.1.1.281 | First |
| hCoV-19/India/MH-ACTREC-368/2020 | betacoronavirus | EPI_ISL_700017 | 24-07-2020 | India | 59 | Female | B.1.1.281 | First |
| hCoV-19/India/MH-ACTREC-369/2020 | betacoronavirus | EPI_ISL_700018 | 24-07-2020 | India | 59 | Male | B.1.1.281 | First |
| hCoV-19/India/MH-ACTREC-370/2020 | betacoronavirus | EPI_ISL_700019 | 24-07-2020 | India | 75 | Male | B.1.1.281 | First |
| hCoV-19/India/MH-ACTREC-371/2020 | betacoronavirus | EPI_ISL_700020 | 24-07-2020 | India | 63 | Male | B.1.1.281 | First |
| hCoV-19/India/MH-ACTREC-372/2020 | betacoronavirus | EPI_ISL_700021 | 24-07-2020 | India | 54 | Female | B.1.1.281 | First |
| hCoV-19/India/MH-ACTREC-373/2020 | betacoronavirus | EPI_ISL_700022 | 24-07-2020 | India | 50 | Female | B.1.1.281 | First |
| hCoV-19/India/MH-ACTREC-374/2020 | betacoronavirus | EPI_ISL_700023 | 24-07-2020 | India | 49 | Male | B.1.1.306 | First |
| hCoV-19/India/MH-ACTREC-375/2020 | betacoronavirus | EPI_ISL_700024 | 24-07-2020 | India | 20 | Female | B.1.1.281 | First |
| hCoV-19/India/MH-ACTREC-376/2020 | betacoronavirus | EPI_ISL_700025 | 24-07-2020 | India | 45 | Female | B.1.1.281 | First |
| hCoV-19/India/MH-ACTREC-377/2020 | betacoronavirus | EPI_ISL_700026 | 24-07-2020 | India | 43 | Male | B.1.1 | First |
| hCoV-19/India/MH-ACTREC-378/2020 | betacoronavirus | EPI_ISL_700027 | 24-07-2020 | India | 43 | Male | B.1.210 | First |
| hCoV-19/India/MH-ACTREC-379/2020 | betacoronavirus | EPI_ISL_700028 | 24-07-2020 | India | 28 | Male | B.1.210 | First |
| hCoV-19/India/MH-ACTREC-380/2020 | betacoronavirus | EPI_ISL_700029 | 25-07-2020 | India | 47 | Male | B.1.1.306 | First |
| hCoV-19/India/MH-ACTREC-381/2020 | betacoronavirus | EPI_ISL_700030 | 25-07-2020 | India | 30 | Female | B.1.247 | First |
| hCoV-19/India/MH-ACTREC-382/2020 | betacoronavirus | EPI_ISL_700031 | 25-07-2020 | India | 52 | Male | B.1.1.281 | First |
| hCoV-19/India/MH-ACTREC-383/2020 | betacoronavirus | EPI_ISL_700032 | 27-07-2020 | India | 46 | Male | B.1.1 | First |
| hCoV-19/India/MH-ACTREC-384/2020 | betacoronavirus | EPI_ISL_700033 | 27-07-2020 | India | 33 | Male | B.1.1.281 | First |
| hCoV-19/India/MH-ACTREC-385/2020 | betacoronavirus | EPI_ISL_700034 | 27-07-2020 | India | 36 | Male | B.1.1.281 | First |
| hCoV-19/India/MH-ACTREC-386/2020 | betacoronavirus | EPI_ISL_700035 | 27-07-2020 | India | 22 | Male | B.1.1.281 | First |
| hCoV-19/India/MH-ACTREC-387/2020 | betacoronavirus | EPI_ISL_700036 | 28-07-2020 | India | 30 | Male | B.1.1.306 | First |
| hCoV-19/India/MH-ACTREC-388/2020 | betacoronavirus | EPI_ISL_700037 | 28-07-2020 | India | 29 | Male | B.1.1.101 | First |
| hCoV-19/India/MH-ACTREC-389/2020 | betacoronavirus | EPI_ISL_700038 | 28-07-2020 | India | 43 | Male | B.1.1.306 | First |
| hCoV-19/India/MH-ACTREC-390/2020 | betacoronavirus | EPI_ISL_700039 | 29-07-2020 | India | 63 | Female | B.1.1.281 | First |
| hCoV-19/India/MH-ACTREC-391/2020 | betacoronavirus | EPI_ISL_700040 | 29-07-2020 | India | 29 | Female | B.1.1.306 | First |
| hCoV-19/India/MH-ACTREC-392/2020 | betacoronavirus | EPI_ISL_700041 | 29-07-2020 | India | 1 | Female | B.1.1.306 | First |
| hCoV-19/India/MH-ACTREC-393/2020 | betacoronavirus | EPI_ISL_700042 | 29-07-2020 | India | 37 | Female | B.1.1.306 | First |
| hCoV-19/India/MH-ACTREC-394/2020 | betacoronavirus | EPI_ISL_700043 | 29-07-2020 | India | 35 | Male | B.1.1.101 | First |
| hCoV-19/India/MH-ACTREC-395/2020 | betacoronavirus | EPI_ISL_700044 | 29-07-2020 | India | 49 | Male | B.1.210 | First |
| hCoV-19/India/MH-ACTREC-396/2020 | betacoronavirus | EPI_ISL_700045 | 29-07-2020 | India | 59 | Male | B.1.5 | First |
| hCoV-19/India/MH-ACTREC-397/2020 | betacoronavirus | EPI_ISL_700046 | 29-07-2020 | India | 40 | Male | B.1.217 | First |
| hCoV-19/India/MH-ACTREC-398/2020 | betacoronavirus | EPI_ISL_700047 | 29-07-2020 | India | 33 | Male | B.1.1.281 | First |
| hCoV-19/India/MH-ACTREC-399/2020 | betacoronavirus | EPI_ISL_700048 | 29-07-2020 | India | 32 | Male | B.1.210 | First |
| hCoV-19/India/MH-ACTREC-400/2020 | betacoronavirus | EPI_ISL_700049 | 29-07-2020 | India | 36 | Female | B.1.217 | First |
| hCoV-19/India/MH-ACTREC-401/2020 | betacoronavirus | EPI_ISL_700050 | 29-07-2020 | India | 26 | Female | B.1.1.281 | First |
| hCoV-19/India/MH-ACTREC-402/2020 | betacoronavirus | EPI_ISL_700051 | 29-07-2020 | India | 28 | Male | B.1.1.306 | First |
| hCoV-19/India/MH-ACTREC-403/2020 | betacoronavirus | EPI_ISL_700052 | 29-07-2020 | India | 37 | Male | B.1.1.281 | First |
| hCoV-19/India/MH-ACTREC-404/2020 | betacoronavirus | EPI_ISL_700053 | 29-07-2020 | India | 66 | Male | B.1.1.281 | First |
| hCoV-19/India/MH-ACTREC-405/2020 | betacoronavirus | EPI_ISL_700054 | 29-07-2020 | India | 34 | Female | B.1.217 | First |
| hCoV-19/India/MH-ACTREC-406/2020 | betacoronavirus | EPI_ISL_700055 | 29-07-2020 | India | 4 | Male | B.1.217 | First |
| hCoV-19/India/MH-ACTREC-407/2020 | betacoronavirus | EPI_ISL_700056 | 29-07-2020 | India | 66 | Male | B.1.217 | First |
| hCoV-19/India/MH-ACTREC-408/2020 | betacoronavirus | EPI_ISL_700057 | 29-07-2020 | India | 29 | Female | B.1.1.101 | First |
| hCoV-19/India/MH-ACTREC-409/2020 | betacoronavirus | EPI_ISL_700058 | 29-07-2020 | India | 68 | Male | B.1.1.101 | First |
| hCoV-19/India/MH-ACTREC-410/2020 | betacoronavirus | EPI_ISL_700059 | 29-07-2020 | India | 39 | Male | B.1.1.101 | First |
| hCoV-19/India/MH-ACTREC-411/2020 | betacoronavirus | EPI_ISL_700060 | 29-07-2020 | India | 42 | Male | B.1.210 | First |
| hCoV-19/India/MH-ACTREC-412/2020 | betacoronavirus | EPI_ISL_700061 | 29-07-2020 | India | 26 | Male | B.1.1.281 | First |
| hCoV-19/India/MH-ACTREC-413/2020 | betacoronavirus | EPI_ISL_700062 | 29-07-2020 | India | 36 | Female | B.1.210 | First |
| hCoV-19/India/MH-ACTREC-414/2020 | betacoronavirus | EPI_ISL_700063 | 30-07-2020 | India | 34 | Male | B.1.1.281 | First |
| hCoV-19/India/MH-ACTREC-415/2020 | betacoronavirus | EPI_ISL_700064 | 30-07-2020 | India | 55 | Male | B.1.1.101 | First |
| hCoV-19/India/MH-ACTREC-416/2020 | betacoronavirus | EPI_ISL_700065 | 30-07-2020 | India | 35 | Female | B.1.210 | First |
| hCoV-19/India/MH-ACTREC-417/2020 | betacoronavirus | EPI_ISL_700066 | 30-07-2020 | India | 44 | Female | B.1.1.212 | First |
| hCoV-19/India/MH-ACTREC-418/2020 | betacoronavirus | EPI_ISL_700067 | 30-07-2020 | India | 60 | Male | B.1.217 | First |
| hCoV-19/India/MH-ACTREC-419/2020 | betacoronavirus | EPI_ISL_700068 | 30-07-2020 | India | 39 | Male | B.1.1.281 | First |
| hCoV-19/India/MH-ACTREC-420/2020 | betacoronavirus | EPI_ISL_700069 | 30-07-2020 | India | 38 | Male | B.1.210 | First |
| hCoV-19/India/UP-ACTREC-421/2020 | betacoronavirus | EPI_ISL_700070 | 31-07-2020 | India | 55 | Female | B.1.1.281 | First |
| hCoV-19/India/MH-ACTREC-422/2020 | betacoronavirus | EPI_ISL_700071 | 31-07-2020 | India | 57 | Male | B.1.1.281 | First |
| hCoV-19/India/MH-ACTREC-423/2020 | betacoronavirus | EPI_ISL_700072 | 31-07-2020 | India | 55 | Female | B.1.210 | First |
| hCoV-19/India/MH-ACTREC-424/2020 | betacoronavirus | EPI_ISL_700073 | 31-07-2020 | India | 38 | Male | B.1.210 | First |
| hCoV-19/India/MH-ACTREC-425/2020 | betacoronavirus | EPI_ISL_700074 | 31-07-2020 | India | 60 | Male | B.1.1.281 | First |
| hCoV-19/India/MH-ACTREC-426/2020 | betacoronavirus | EPI_ISL_700075 | 31-07-2020 | India | 42 | Male | B.1.1.281 | First |
| hCoV-19/India/MH-ACTREC-427/2020 | betacoronavirus | EPI_ISL_700076 | 31-07-2020 | India | 34 | Male | B.1.1.281 | First |
| hCoV-19/India/MH-ACTREC-428/2020 | betacoronavirus | EPI_ISL_700077 | 31-07-2020 | India | 61 | Female | B.1.1 | First |
| hCoV-19/India/MH-ACTREC-429/2020 | betacoronavirus | EPI_ISL_700078 | 31-07-2020 | India | 49 | Male | B.1.1.306 | First |
| hCoV-19/India/MH-ACTREC-430/2020 | betacoronavirus | EPI_ISL_700079 | 04-08-2020 | India | 26 | Male | B.1.1.306 | First |
| hCoV-19/India/MH-ACTREC-431/2020 | betacoronavirus | EPI_ISL_700080 | 04-08-2020 | India | 55 | Male | B.1.1 | First |
| hCoV-19/India/MH-ACTREC-432/2020 | betacoronavirus | EPI_ISL_700081 | 04-08-2020 | India | 40 | Male | B.1.210 | First |
| hCoV-19/India/MH-ACTREC-433/2020 | betacoronavirus | EPI_ISL_700082 | 04-08-2020 | India | 31 | Female | B.1.1.306 | First |
| hCoV-19/India/MH-ACTREC-434/2020 | betacoronavirus | EPI_ISL_700083 | 04-08-2020 | India | 53 | Female | B.1.1.281 | First |
| hCoV-19/India/MH-ACTREC-435/2020 | betacoronavirus | EPI_ISL_700084 | 04-08-2020 | India | 43 | Male | B.1.1.101 | First |
| hCoV-19/India/MH-ACTREC-436/2020 | betacoronavirus | EPI_ISL_700085 | 05-08-2020 | India | 26 | Female | B.1.1.281 | First |
| hCoV-19/India/MH-ACTREC-437/2020 | betacoronavirus | EPI_ISL_700086 | 05-08-2020 | India | 35 | Male | B.1.210 | First |
| hCoV-19/India/MH-ACTREC-438/2020 | betacoronavirus | EPI_ISL_700087 | 05-08-2020 | India | 62 | Female | B.1.1.281 | First |
| hCoV-19/India/MH-ACTREC-439/2020 | betacoronavirus | EPI_ISL_700088 | 05-08-2020 | India | 38 | Male | B.1.217 | First |
| hCoV-19/India/MH-ACTREC-440/2020 | betacoronavirus | EPI_ISL_700089 | 05-08-2020 | India | 34 | Female | B.1.1.281 | First |
| hCoV-19/India/MH-ACTREC-441/2020 | betacoronavirus | EPI_ISL_700090 | 05-08-2020 | India | 65 | Female | B.1.210 | First |
| hCoV-19/India/MH-ACTREC-442/2020 | betacoronavirus | EPI_ISL_700091 | 05-08-2020 | India | 44 | Female | B.1.1.281 | First |
| hCoV-19/India/MH-ACTREC-443/2020 | betacoronavirus | EPI_ISL_723053 | 05-08-2020 | India | 38 | Male | B.1.1.212 | First |
| hCoV-19/India/MH-ACTREC-444/2020 | betacoronavirus | EPI_ISL_700092 | 05-08-2020 | India | 40 | Female | B.1.210 | First |
| hCoV-19/India/MH-ACTREC-445/2020 | betacoronavirus | EPI_ISL_700093 | 05-08-2020 | India | 42 | Male | B.1.1.306 | First |
| hCoV-19/India/MH-ACTREC-446/2020 | betacoronavirus | EPI_ISL_700094 | 05-08-2020 | India | 29 | Female | B.1.1.306 | First |
| hCoV-19/India/MH-ACTREC-447/2020 | betacoronavirus | EPI_ISL_700095 | 07-08-2020 | India | 29 | Male | B.1.210 | First |
| hCoV-19/India/MH-ACTREC-448/2020 | betacoronavirus | EPI_ISL_700096 | 07-08-2020 | India | 19 | Male | B.1.1.281 | First |
| hCoV-19/India/MH-ACTREC-449/2020 | betacoronavirus | EPI_ISL_700097 | 07-08-2020 | India | 59 | Male | B.1.1.281 | First |
| hCoV-19/India/MH-ACTREC-450/2020 | betacoronavirus | EPI_ISL_700098 | 07-08-2020 | India | 50 | Female | B.1.1.281 | First |
| hCoV-19/India/MH-ACTREC-451/2020 | betacoronavirus | EPI_ISL_700099 | 07-08-2020 | India | 55 | Male | B.1.1.281 | First |
| hCoV-19/India/MH-ACTREC-452/2020 | betacoronavirus | EPI_ISL_700100 | 08-08-2020 | India | 36 | Female | B.1.210 | First |
| hCoV-19/India/MH-ACTREC-453/2020 | betacoronavirus | EPI_ISL_700101 | 08-08-2020 | India | 12 | Female | B.1.210 | First |
| hCoV-19/India/MH-ACTREC-454/2020 | betacoronavirus | EPI_ISL_700102 | 09-08-2020 | India | 31 | Male | B.1.210 | First |
| hCoV-19/India/MH-ACTREC-455/2020 | betacoronavirus | EPI_ISL_700103 | 09-08-2020 | India | 66 | Male | B.1.1.281 | First |
| hCoV-19/India/MH-ACTREC-456/2020 | betacoronavirus | EPI_ISL_700104 | 09-08-2020 | India | 35 | Male | B.1.1.281 | First |
| hCoV-19/India/MH-ACTREC-457/2020 | betacoronavirus | EPI_ISL_700105 | 09-08-2020 | India | 37 | Male | B.1.1.281 | First |
| hCoV-19/India/MH-ACTREC-458/2020 | betacoronavirus | EPI_ISL_700106 | 09-08-2020 | India | 30 | Female | B.1.217 | First |
| hCoV-19/India/MH-ACTREC-459/2020 | betacoronavirus | EPI_ISL_700107 | 10-08-2020 | India | 66 | Male | B.1.210 | First |
| hCoV-19/India/MH-ACTREC-460/2020 | betacoronavirus | EPI_ISL_700108 | 10-08-2020 | India | 23 | Female | B.1.1.281 | First |
| hCoV-19/India/MH-ACTREC-461/2020 | betacoronavirus | EPI_ISL_700109 | 10-08-2020 | India | 56 | Female | B.1.1.281 | First |
| hCoV-19/India/MH-ACTREC-462/2020 | betacoronavirus | EPI_ISL_700110 | 10-08-2020 | India | 50 | Male | B.1.1.101 | First |
| hCoV-19/India/MH-ACTREC-463/2020 | betacoronavirus | EPI_ISL_700111 | 10-08-2020 | India | 43 | Male | B.1.210 | First |
| hCoV-19/India/MH-ACTREC-464/2020 | betacoronavirus | EPI_ISL_700112 | 10-08-2020 | India | 35 | Male | B.1.1.281 | First |
| hCoV-19/India/MH-ACTREC-465/2020 | betacoronavirus | EPI_ISL_700113 | 10-08-2020 | India | 60 | Male | B.1.1.306 | First |
| hCoV-19/India/MH-ACTREC-466/2020 | betacoronavirus | EPI_ISL_700114 | 10-08-2020 | India | 42 | Male | B.1.1.281 | First |
| hCoV-19/India/MH-ACTREC-467/2020 | betacoronavirus | EPI_ISL_700115 | 10-08-2020 | India | 67 | Female | B.1.217 | First |
| hCoV-19/India/MH-ACTREC-468/2020 | betacoronavirus | EPI_ISL_700116 | 10-08-2020 | India | 30 | Male | B.1.1.281 | First |
| hCoV-19/India/MH-ACTREC-469/2020 | betacoronavirus | EPI_ISL_700117 | 11-08-2020 | India | 49 | Male | B.1.210 | First |
| hCoV-19/India/MH-ACTREC-470/2020 | betacoronavirus | EPI_ISL_700118 | 11-08-2020 | India | 62 | Male | B.1.1.281 | First |
| hCoV-19/India/MH-ACTREC-471/2020 | betacoronavirus | EPI_ISL_700119 | 11-08-2020 | India | 30 | Female | B.1.210 | First |
| hCoV-19/India/MH-ACTREC-472/2020 | betacoronavirus | EPI_ISL_700120 | 11-08-2020 | India | 66 | Male | B.1.210 | First |
| hCoV-19/India/MH-ACTREC-473/2020 | betacoronavirus | EPI_ISL_700121 | 11-08-2020 | India | 18 | Female | B.1.210 | First |
| hCoV-19/India/MH-ACTREC-474/2020 | betacoronavirus | EPI_ISL_700122 | 11-08-2020 | India | 38 | Male | B.1.1.281 | First |
| hCoV-19/India/MH-ACTREC-475/2020 | betacoronavirus | EPI_ISL_700123 | 11-08-2020 | India | 35 | Female | B.1.210 | First |
| hCoV-19/India/MH-ACTREC-476/2020 | betacoronavirus | EPI_ISL_700124 | 11-08-2020 | India | 34 | Male | B.1.210 | First |
| hCoV-19/India/MH-ACTREC-477/2020 | betacoronavirus | EPI_ISL_700125 | 11-08-2020 | India | 26 | Male | B.1.1.179 | First |
| hCoV-19/India/MH-ACTREC-478/2020 | betacoronavirus | EPI_ISL_700126 | 11-08-2020 | India | 35 | Male | B.1.1.281 | First |
| hCoV-19/India/MH-ACTREC-479/2020 | betacoronavirus | EPI_ISL_700127 | 11-08-2020 | India | 50 | Female | B.1.1.281 | First |
| hCoV-19/India/MH-ACTREC-480/2020 | betacoronavirus | EPI_ISL_700128 | 12-08-2020 | India | 27 | Male | B.1 | First |
| hCoV-19/India/MH-ACTREC-481/2020 | betacoronavirus | EPI_ISL_700129 | 13-08-2020 | India | 55 | Female | B.1.210 | First |
| hCoV-19/India/MH-ACTREC-482/2020 | betacoronavirus | EPI_ISL_700130 | 13-08-2020 | India | 42 | Male | B.1.210 | First |
| hCoV-19/India/MH-ACTREC-483/2020 | betacoronavirus | EPI_ISL_700131 | 13-08-2020 | India | 46 | Female | B.1.210 | First |
| hCoV-19/India/MH-ACTREC-484/2020 | betacoronavirus | EPI_ISL_700132 | 13-08-2020 | India | 25 | Female | B.1.210 | First |
| hCoV-19/India/MH-ACTREC-485/2020 | betacoronavirus | EPI_ISL_700133 | 13-08-2020 | India | 38 | Male | B.1.210 | First |
| hCoV-19/India/MH-ACTREC-486/2020 | betacoronavirus | EPI_ISL_700134 | 13-08-2020 | India | 13 | Male | B.1.217 | First |
| hCoV-19/India/MH-ACTREC-487/2020 | betacoronavirus | EPI_ISL_700135 | 13-08-2020 | India | 32 | Female | B.1.217 | First |
| hCoV-19/India/MH-ACTREC-488/2020 | betacoronavirus | EPI_ISL_700136 | 13-08-2020 | India | 28 | Female | B.1.1.281 | First |
| hCoV-19/India/MH-ACTREC-489/2020 | betacoronavirus | EPI_ISL_700137 | 13-08-2020 | India | 44 | Male | B.1.1.281 | First |
| hCoV-19/India/MH-ACTREC-490/2020 | betacoronavirus | EPI_ISL_700138 | 13-08-2020 | India | 59 | Male | B.1.36 | First |
| hCoV-19/India/MH-ACTREC-491/2020 | betacoronavirus | EPI_ISL_700139 | 14-08-2020 | India | 23 | Male | B.1.1.212 | First |
| hCoV-19/India/MH-ACTREC-492/2020 | betacoronavirus | EPI_ISL_700140 | 14-08-2020 | India | 43 | Male | B.1.1.306 | First |
| hCoV-19/India/MH-ACTREC-493/2020 | betacoronavirus | EPI_ISL_700141 | 14-08-2020 | India | 26 | Male | B.1.210 | First |
| hCoV-19/India/MH-ACTREC-494/2020 | betacoronavirus | EPI_ISL_700142 | 14-08-2020 | India | 22 | Male | B.1.1.281 | First |
| hCoV-19/India/MH-ACTREC-495/2020 | betacoronavirus | EPI_ISL_700143 | 14-08-2020 | India | 67 | Male | B.1.1.281 | First |
| hCoV-19/India/MH-ACTREC-496/2020 | betacoronavirus | EPI_ISL_700144 | 14-08-2020 | India | 50 | Male | B.1.1.212 | First |
| hCoV-19/India/MH-ACTREC-497/2020 | betacoronavirus | EPI_ISL_700145 | 14-08-2020 | India | 62 | Male | B.1.1.281 | First |
| hCoV-19/India/MH-ACTREC-498/2020 | betacoronavirus | EPI_ISL_700146 | 14-08-2020 | India | 55 | Female | B.1.1.281 | First |
| hCoV-19/India/MH-ACTREC-499/2020 | betacoronavirus | EPI_ISL_700147 | 14-08-2020 | India | 87 | Male | B.1.1.306 | First |
| hCoV-19/India/MH-ACTREC-500/2020 | betacoronavirus | EPI_ISL_700148 | 14-08-2020 | India | 44 | Male | B.1.1.281 | First |
| hCoV-19/India/MH-ACTREC-501/2020 | betacoronavirus | EPI_ISL_700149 | 15-08-2020 | India | 33 | Female | B.1.1.306 | First |
| hCoV-19/India/MH-ACTREC-502/2020 | betacoronavirus | EPI_ISL_700150 | 15-08-2020 | India | 35 | Female | B.1.210 | First |
| hCoV-19/India/MH-ACTREC-503/2020 | betacoronavirus | EPI_ISL_700151 | 15-08-2020 | India | 15 | Female | B.1.210 | First |
| hCoV-19/India/MH-ACTREC-504/2020 | betacoronavirus | EPI_ISL_700152 | 17-08-2020 | India | 33 | Male | B.1.1.281 | First |
| hCoV-19/India/MH-ACTREC-505/2020 | betacoronavirus | EPI_ISL_700153 | 17-08-2020 | India | 31 | Female | B.1.1.306 | First |
| hCoV-19/India/MH-ACTREC-506/2020 | betacoronavirus | EPI_ISL_700154 | 17-08-2020 | India | 46 | Male | B.1.210 | First |
| hCoV-19/India/MH-ACTREC-507/2020 | betacoronavirus | EPI_ISL_700155 | 17-08-2020 | India | 33 | Male | B.1.210 | First |
| hCoV-19/India/MH-ACTREC-508/2020 | betacoronavirus | EPI_ISL_700156 | 18-08-2020 | India | 62 | Male | B.1.1.281 | First |
| hCoV-19/India/MH-ACTREC-509/2020 | betacoronavirus | EPI_ISL_700157 | 18-08-2020 | India | 52 | Female | B.1.1.306 | First |
| hCoV-19/India/MH-ACTREC-510/2020 | betacoronavirus | EPI_ISL_700158 | 19-08-2020 | India | 24 | Female | B.1.1 | First |
| hCoV-19/India/MH-ACTREC-511/2020 | betacoronavirus | EPI_ISL_700159 | 19-08-2020 | India | 39 | Female | B.1.1.306 | First |
| hCoV-19/India/MH-ACTREC-512/2020 | betacoronavirus | EPI_ISL_723054 | 19-08-2020 | India | 38 | Male | B.1.1.306 | First |
| hCoV-19/India/MH-ACTREC-513/2020 | betacoronavirus | EPI_ISL_700160 | 19-08-2020 | India | 36 | Male | B.1.1.306 | First |
| hCoV-19/India/MH-ACTREC-514/2020 | betacoronavirus | EPI_ISL_700161 | 19-08-2020 | India | 24 | Female | B.1 | First |
| hCoV-19/India/MH-ACTREC-515/2020 | betacoronavirus | EPI_ISL_700162 | 19-08-2020 | India | 52 | Female | B.1.5 | First |
| hCoV-19/India/MH-ACTREC-516/2020 | betacoronavirus | EPI_ISL_700163 | 19-08-2020 | India | 21 | Male | B.1.1.281 | First |
| hCoV-19/India/MH-ACTREC-517/2020 | betacoronavirus | EPI_ISL_723055 | 20-08-2020 | India | 70 | Male | B.1.1.306 | First |
| hCoV-19/India/MH-ACTREC-518/2020 | betacoronavirus | EPI_ISL_700164 | 21-08-2020 | India | 55 | Female | B.1.1.306 | First |
| hCoV-19/India/MH-ACTREC-519/2020 | betacoronavirus | EPI_ISL_700165 | 21-08-2020 | India | 45 | Male | B.1.1.306 | First |
| hCoV-19/India/MH-ACTREC-520/2020 | betacoronavirus | EPI_ISL_700166 | 21-08-2020 | India | 45 | Male | B.1.1.306 | First |
| hCoV-19/India/MH-ACTREC-521/2020 | betacoronavirus | EPI_ISL_700167 | 21-08-2020 | India | 27 | Female | B.1.1.306 | First |
| hCoV-19/India/MH-ACTREC-522/2020 | betacoronavirus | EPI_ISL_700168 | 21-08-2020 | India | 26 | Female | B.1.1 | First |
| hCoV-19/India/MH-ACTREC-523/2020 | betacoronavirus | EPI_ISL_700169 | 21-08-2020 | India | 50 | Female | B.1.1 | First |
| hCoV-19/India/MH-ACTREC-524/2020 | betacoronavirus | EPI_ISL_700170 | 22-08-2020 | India | 59 | Female | B.1.1.281 | First |
| hCoV-19/India/MH-ACTREC-525/2020 | betacoronavirus | EPI_ISL_700171 | 22-08-2020 | India | 51 | Male | B.1.210 | First |
| hCoV-19/India/MH-ACTREC-526/2020 | betacoronavirus | EPI_ISL_700172 | 22-08-2020 | India | 21 | Male | B.1.1 | First |
| hCoV-19/India/MH-ACTREC-527/2020 | betacoronavirus | EPI_ISL_700173 | 22-08-2020 | India | 29 | Male | B.1.1.8 | First |
| hCoV-19/India/MH-ACTREC-528/2020 | betacoronavirus | EPI_ISL_700174 | 24-08-2020 | India | 35 | Male | B.1.1 | First |
| hCoV-19/India/MH-ACTREC-529/2020 | betacoronavirus | EPI_ISL_700175 | 25-08-2020 | India | 26 | Male | B.1.1.306 | First |
| hCoV-19/India/MH-ACTREC-530/2020 | betacoronavirus | EPI_ISL_700176 | 25-08-2020 | India | 21 | Male | B.1.1.306 | First |
| hCoV-19/India/MH-ACTREC-531/2020 | betacoronavirus | EPI_ISL_700177 | 25-08-2020 | India | 25 | Male | B.1.1.306 | First |
| hCoV-19/India/MH-ACTREC-532/2020 | betacoronavirus | EPI_ISL_700178 | 25-08-2020 | India | 46 | Male | B.1.1.281 | First |
| hCoV-19/India/MH-ACTREC-533/2020 | betacoronavirus | EPI_ISL_700179 | 25-08-2020 | India | 41 | Male | B.1.1.281 | First |
| hCoV-19/India/MH-ACTREC-534/2020 | betacoronavirus | EPI_ISL_700180 | 25-08-2020 | India | 64 | Female | B.1.1.281 | First |
| hCoV-19/India/MH-ACTREC-535/2020 | betacoronavirus | EPI_ISL_700181 | 25-08-2020 | India | 55 | Female | B.1.1.281 | First |
| hCoV-19/India/MH-ACTREC-536/2020 | betacoronavirus | EPI_ISL_700182 | 25-08-2020 | India | 38 | Male | B.1.1.281 | First |
| hCoV-19/India/MH-ACTREC-537/2020 | betacoronavirus | EPI_ISL_700183 | 25-08-2020 | India | 38 | Male | B.1.210 | First |
| hCoV-19/India/MH-ACTREC-538/2020 | betacoronavirus | EPI_ISL_700184 | 25-08-2020 | India | 57 | Male | B.1.1.306 | First |
| hCoV-19/India/MH-ACTREC-539/2020 | betacoronavirus | EPI_ISL_700185 | 25-08-2020 | India | 39 | Male | B.1.1.281 | First |
| hCoV-19/India/MH-ACTREC-540/2020 | betacoronavirus | EPI_ISL_700186 | 26-08-2020 | India | 67 | Male | B.1.1.306 | First |
| hCoV-19/India/MH-ACTREC-541/2020 | betacoronavirus | EPI_ISL_700187 | 26-08-2020 | India | 28 | Male | B.1.1.281 | First |
| hCoV-19/India/MH-ACTREC-542/2020 | betacoronavirus | EPI_ISL_700188 | 26-08-2020 | India | 25 | Male | B.1 | First |
| hCoV-19/India/MH-ACTREC-543/2020 | betacoronavirus | EPI_ISL_700189 | 26-08-2020 | India | 58 | Male | B.1.1.281 | First |
| hCoV-19/India/MH-ACTREC-544/2020 | betacoronavirus | EPI_ISL_700190 | 26-08-2020 | India | 25 | Male | B.1.1 | First |
| hCoV-19/India/MH-ACTREC-545/2020 | betacoronavirus | EPI_ISL_700191 | 27-08-2020 | India | 56 | Female | B.1.1 | First |
| hCoV-19/India/MH-ACTREC-546/2020 | betacoronavirus | EPI_ISL_723056 | 27-08-2020 | India | 50 | Female | B.1.1.281 | First |
| hCoV-19/India/MH-ACTREC-547/2020 | betacoronavirus | EPI_ISL_700192 | 27-08-2020 | India | 36 | Male | B.1.1.281 | First |
| hCoV-19/India/MH-ACTREC-548/2020 | betacoronavirus | EPI_ISL_700193 | 28-08-2020 | India | 50 | Male | B.1.1.101 | First |
| hCoV-19/India/MH-ACTREC-549/2020 | betacoronavirus | EPI_ISL_700194 | 28-08-2020 | India | 32 | Female | B.1.1.216 | First |
| hCoV-19/India/MH-ACTREC-550/2020 | betacoronavirus | EPI_ISL_700195 | 28-08-2020 | India | 25 | Male | B.1.36.1 | First |
| hCoV-19/India/MH-ACTREC-551/2020 | betacoronavirus | EPI_ISL_700196 | 28-08-2020 | India | 53 | Male | B.1.1.32 | First |
| hCoV-19/India/MH-ACTREC-552/2020 | betacoronavirus | EPI_ISL_700197 | 28-08-2020 | India | 55 | Male | B.1.1.281 | First |
| hCoV-19/India/MH-ACTREC-553/2020 | betacoronavirus | EPI_ISL_700198 | 28-08-2020 | India | 32 | Male | B.1.210 | First |
| hCoV-19/India/MH-ACTREC-554/2020 | betacoronavirus | EPI_ISL_700199 | 28-08-2020 | India | 28 | Female | B.1.1.226 | First |
| hCoV-19/India/MH-ACTREC-555/2020 | betacoronavirus | EPI_ISL_700200 | 28-08-2020 | India | 80 | Male | B.1.1.8 | First |
| hCoV-19/India/MH-ACTREC-556/2020 | betacoronavirus | EPI_ISL_700201 | 28-08-2020 | India | 49 | Male | B.1.1.281 | First |
| hCoV-19/India/MH-ACTREC-557/2020 | betacoronavirus | EPI_ISL_700202 | 29-08-2020 | India | 41 | Female | B.1.247 | First |
| hCoV-19/India/MH-ACTREC-558/2020 | betacoronavirus | EPI_ISL_700203 | 29-08-2020 | India | 55 | Male | B.1.210 | First |
| hCoV-19/India/MH-ACTREC-559/2020 | betacoronavirus | EPI_ISL_700204 | 29-08-2020 | India | 39 | Male | B.1.1 | First |
| hCoV-19/India/MH-ACTREC-560/2020 | betacoronavirus | EPI_ISL_700205 | 29-08-2020 | India | 13 | Male | B.1.1.306 | First |
| hCoV-19/India/MH-ACTREC-561/2020 | betacoronavirus | EPI_ISL_700206 | 29-08-2020 | India | 20 | Male | B.1 | First |
| hCoV-19/India/MH-ACTREC-562/2020 | betacoronavirus | EPI_ISL_700207 | 31-08-2020 | India | 26 | Male | B.1.1.281 | First |
| hCoV-19/India/MH-ACTREC-563/2020 | betacoronavirus | EPI_ISL_700208 | 31-08-2020 | India | 26 | Male | B.1.217 | First |
| hCoV-19/India/MH-ACTREC-564/2020 | betacoronavirus | EPI_ISL_700209 | 31-08-2020 | India | 15 | Male | B.1.1.281 | First |
| hCoV-19/India/MH-ACTREC-565/2020 | betacoronavirus | EPI_ISL_700210 | 31-08-2020 | India | 43 | Female | B.1.1.281 | First |
| hCoV-19/India/MH-ACTREC-566/2020 | betacoronavirus | EPI_ISL_723057 | 31-08-2020 | India | 46 | Male | B.1.36.6 | First |
| hCoV-19/India/MH-ACTREC-567/2020 | betacoronavirus | EPI_ISL_700211 | 31-08-2020 | India | 33 | Female | B.1.1.281 | First |
| hCoV-19/India/MH-ACTREC-568/2020 | betacoronavirus | EPI_ISL_700212 | 31-08-2020 | India | 35 | Male | B.1.1.281 | First |
| hCoV-19/India/MH-ACTREC-569/2020 | betacoronavirus | EPI_ISL_700213 | 31-08-2020 | India | 23 | Male | B.1.1 | First |
| hCoV-19/India/MH-ACTREC-570/2020 | betacoronavirus | EPI_ISL_700214 | 31-08-2020 | India | 20 | Female | B.1.1.281 | First |
| hCoV-19/India/MH-ACTREC-571/2020 | betacoronavirus | EPI_ISL_700215 | 31-08-2020 | India | 42 | Male | B.1.1.306 | First |
| hCoV-19/India/MH-ACTREC-572/2020 | betacoronavirus | EPI_ISL_700216 | 31-08-2020 | India | 48 | Male | B.1.217 | First |
| hCoV-19/India/MH-ACTREC-573/2020 | betacoronavirus | EPI_ISL_700217 | 31-08-2020 | India | 25 | Male | B.1.1.216 | First |
| hCoV-19/India/MH-ACTREC-574/2020 | betacoronavirus | EPI_ISL_700218 | 01-09-2020 | India | 49 | Male | B.1.210 | First |
| hCoV-19/India/MH-ACTREC-575/2020 | betacoronavirus | EPI_ISL_700219 | 01-09-2020 | India | 32 | Male | B.1.1.281 | First |
| hCoV-19/India/MH-ACTREC-576/2020 | betacoronavirus | EPI_ISL_700220 | 02-09-2020 | India | 45 | Female | B.1.1.306 | First |
| hCoV-19/India/MH-ACTREC-577/2020 | betacoronavirus | EPI_ISL_723058 | 02-09-2020 | India | 30 | Male | B.1.1.281 | First |
| hCoV-19/India/MH-ACTREC-578/2020 | betacoronavirus | EPI_ISL_700221 | 02-09-2020 | India | 60 | Female | B.1.1.101 | First |
| hCoV-19/India/MH-ACTREC-579/2020 | betacoronavirus | EPI_ISL_723059 | 02-09-2020 | India | 37 | Male | B.1.210 | First |
| hCoV-19/India/MH-ACTREC-580/2020 | betacoronavirus | EPI_ISL_700222 | 02-09-2020 | India | 30 | Female | B.1.210 | First |
| hCoV-19/India/MH-ACTREC-581/2020 | betacoronavirus | EPI_ISL_723060 | 02-09-2020 | India | 50 | Male | B.1.210 | First |
| hCoV-19/India/MH-ACTREC-582/2020 | betacoronavirus | EPI_ISL_700223 | 02-09-2020 | India | 51 | Male | B.1.1.212 | First |
| hCoV-19/India/MH-ACTREC-583/2020 | betacoronavirus | EPI_ISL_723061 | 02-09-2020 | India | 38 | Male | B.1.1.212 | First |
| hCoV-19/India/MH-ACTREC-584/2020 | betacoronavirus | EPI_ISL_723062 | 02-09-2020 | India | 36 | Male | B.1.1.212 | First |
| hCoV-19/India/MH-ACTREC-585/2020 | betacoronavirus | EPI_ISL_700224 | 02-09-2020 | India | 34 | Female | B.1.1.281 | First |
| hCoV-19/India/MH-ACTREC-586/2020 | betacoronavirus | EPI_ISL_700225 | 03-09-2020 | India | 31 | Male | B.1.1 | First |
| hCoV-19/India/MH-ACTREC-587/2020 | betacoronavirus | EPI_ISL_700226 | 03-09-2020 | India | 31 | Male | B.1.1.281 | First |
| hCoV-19/India/MH-ACTREC-588/2020 | betacoronavirus | EPI_ISL_700227 | 04-09-2020 | India | 41 | Male | B.1.210 | First |
| hCoV-19/India/MH-ACTREC-589/2020 | betacoronavirus | EPI_ISL_723063 | 04-09-2020 | India | 53 | Female | B.1.1.281 | First |
| hCoV-19/India/MH-ACTREC-590/2020 | betacoronavirus | EPI_ISL_700228 | 04-09-2020 | India | 32 | Male | B.1.1.281 | First |
| hCoV-19/India/MH-ACTREC-591/2020 | betacoronavirus | EPI_ISL_700229 | 04-09-2020 | India | 34 | Male | B.1.36 | First |
| hCoV-19/India/MH-ACTREC-592/2020 | betacoronavirus | EPI_ISL_700230 | 04-09-2020 | India | 39 | Male | B.1.1.281 | First |
| hCoV-19/India/MH-ACTREC-593/2020 | betacoronavirus | EPI_ISL_700231 | 04-09-2020 | India | 61 | Male | B.1.210 | First |
| hCoV-19/India/MH-ACTREC-594/2020 | betacoronavirus | EPI_ISL_700232 | 04-09-2020 | India | 52 | Male | B.1.210 | First |
| hCoV-19/India/MH-ACTREC-595/2020 | betacoronavirus | EPI_ISL_700233 | 05-09-2020 | India | 58 | Male | B.1.36 | First |
| hCoV-19/India/MH-ACTREC-596/2020 | betacoronavirus | EPI_ISL_700234 | 05-09-2020 | India | 8 | Female | B.1.210 | First |
| hCoV-19/India/MH-ACTREC-597/2020 | betacoronavirus | EPI_ISL_700235 | 06-09-2020 | India | 30 | Female | B.1.210 | First |
| hCoV-19/India/MH-ACTREC-598/2020 | betacoronavirus | EPI_ISL_700236 | 07-09-2020 | India | 27 | Male | B.1.1 | First |
| hCoV-19/India/MH-ACTREC-599/2020 | betacoronavirus | EPI_ISL_700237 | 07-09-2020 | India | 72 | Male | B.1.1 | First |
| hCoV-19/India/MH-ACTREC-600/2020 | betacoronavirus | EPI_ISL_700238 | 08-09-2020 | India | 24 | Female | B.1.1.281 | First |
| hCoV-19/India/MH-ACTREC-601/2020 | betacoronavirus | EPI_ISL_700239 | 08-09-2020 | India | 29 | Male | B.1.247 | First |
| hCoV-19/India/MH-ACTREC-602/2020 | betacoronavirus | EPI_ISL_723064 | 08-09-2020 | India | 43 | Male | B.1.1.281 | First |
| hCoV-19/India/MH-ACTREC-603/2020 | betacoronavirus | EPI_ISL_700240 | 08-09-2020 | India | 50 | Male | B.1.217 | First |
| hCoV-19/India/MH-ACTREC-604/2020 | betacoronavirus | EPI_ISL_700241 | 08-09-2020 | India | 66 | Male | B.1.36 | First |
| hCoV-19/India/MH-ACTREC-605/2020 | betacoronavirus | EPI_ISL_700242 | 08-09-2020 | India | 38 | Male | B.1.210 | First |
| hCoV-19/India/MH-ACTREC-606/2020 | betacoronavirus | EPI_ISL_700243 | 09-09-2020 | India | 28 | Male | B.1.210 | First |
| hCoV-19/India/MH-ACTREC-607/2020 | betacoronavirus | EPI_ISL_700244 | 09-09-2020 | India | 51 | Male | B.1.1.306 | First |
| hCoV-19/India/MH-ACTREC-608/2020 | betacoronavirus | EPI_ISL_700245 | 09-09-2020 | India | 45 | Male | B.1.1.306 | First |
| hCoV-19/India/MH-ACTREC-609/2020 | betacoronavirus | EPI_ISL_700246 | 09-09-2020 | India | 62 | Male | B.1.1.306 | First |
| hCoV-19/India/MH-ACTREC-610/2020 | betacoronavirus | EPI_ISL_700247 | 09-09-2020 | India | 56 | Female | B.1.1.306 | First |
| hCoV-19/India/MH-ACTREC-611/2020 | betacoronavirus | EPI_ISL_700248 | 09-09-2020 | India | 60 | Female | B.1.1.306 | First |
| hCoV-19/India/MH-ACTREC-612/2020 | betacoronavirus | EPI_ISL_700249 | 09-09-2020 | India | 8 | Female | B.1.1.306 | First |
| hCoV-19/India/MH-ACTREC-613/2020 | betacoronavirus | EPI_ISL_700250 | 09-09-2020 | India | 12 | Female | B.1.1 | First |
| hCoV-19/India/MH-ACTREC-614/2020 | betacoronavirus | EPI_ISL_723065 | 09-09-2020 | India | 60 | Male | B.1.1.212 | First |
| hCoV-19/India/MH-ACTREC-615/2020 | betacoronavirus | EPI_ISL_723066 | 09-09-2020 | India | 65 | Male | B.1.1.212 | First |
| hCoV-19/India/MH-ACTREC-616/2020 | betacoronavirus | EPI_ISL_700251 | 10-09-2020 | India | 46 | Female | B.1.1.273 | First |
| hCoV-19/India/MH-ACTREC-617/2020 | betacoronavirus | EPI_ISL_700252 | 10-09-2020 | India | 28 | Male | B.1.1.306 | First |
| hCoV-19/India/MH-ACTREC-618/2020 | betacoronavirus | EPI_ISL_700253 | 10-09-2020 | India | 26 | Female | B.1.210 | First |
| hCoV-19/India/MH-ACTREC-619/2020 | betacoronavirus | EPI_ISL_700254 | 10-09-2020 | India | 24 | Female | B.1.1.306 | First |
| hCoV-19/India/MH-ACTREC-620/2020 | betacoronavirus | EPI_ISL_700255 | 11-09-2020 | India | 30 | Male | B.1.217 | First |
| hCoV-19/India/MH-ACTREC-621/2020 | betacoronavirus | EPI_ISL_700256 | 11-09-2020 | India | 36 | Female | B.1.1.281 | First |
| hCoV-19/India/MH-ACTREC-622/2020 | betacoronavirus | EPI_ISL_700257 | 11-09-2020 | India | 58 | Male | B.1.1.306 | First |
| hCoV-19/India/MH-ACTREC-623/2020 | betacoronavirus | EPI_ISL_700258 | 11-09-2020 | India | 70 | Female | B.1.1 | First |
| hCoV-19/India/MH-ACTREC-624/2020 | betacoronavirus | EPI_ISL_700259 | 11-09-2020 | India | 48 | Male | B.1.1 | First |
| hCoV-19/India/MH-ACTREC-625/2020 | betacoronavirus | EPI_ISL_700260 | 11-09-2020 | India | 31 | Female | B.1.36 | First |
| hCoV-19/India/MH-ACTREC-626/2020 | betacoronavirus | EPI_ISL_700261 | 11-09-2020 | India | 28 | Male | B.1.36.1 | First |
| hCoV-19/India/MH-ACTREC-627/2020 | betacoronavirus | EPI_ISL_700262 | 11-09-2020 | India | 32 | Male | B.1.1.281 | First |
| hCoV-19/India/MH-ACTREC-628/2020 | betacoronavirus | EPI_ISL_700263 | 11-09-2020 | India | 29 | Male | B.1.1.281 | First |
| hCoV-19/India/MH-ACTREC-629/2020 | betacoronavirus | EPI_ISL_700264 | 11-09-2020 | India | 35 | Female | B.1.136 | First |
| hCoV-19/India/MH-ACTREC-630/2020 | betacoronavirus | EPI_ISL_723067 | 11-09-2020 | India | 24 | Male | B.1.178 | First |
| hCoV-19/India/MH-ACTREC-631/2020 | betacoronavirus | EPI_ISL_723068 | 11-09-2020 | India | 44 | Male | B.1.210 | First |
| hCoV-19/India/MH-ACTREC-632/2020 | betacoronavirus | EPI_ISL_700265 | 12-09-2020 | India | 57 | Female | B.1.1 | First |
| hCoV-19/India/MH-ACTREC-633/2020 | betacoronavirus | EPI_ISL_700266 | 12-09-2020 | India | 35 | Male | B.1.161 | First |
| hCoV-19/India/MH-ACTREC-634/2020 | betacoronavirus | EPI_ISL_700267 | 12-09-2020 | India | 49 | Female | B.1.36 | First |
| hCoV-19/India/MH-ACTREC-635/2020 | betacoronavirus | EPI_ISL_700268 | 12-09-2020 | India | 41 | Male | B.1.210 | First |
| hCoV-19/India/MH-ACTREC-636/2020 | betacoronavirus | EPI_ISL_700269 | 12-09-2020 | India | 41 | Male | B.1.217 | First |
| hCoV-19/India/MH-ACTREC-637/2020 | betacoronavirus | EPI_ISL_700270 | 13-09-2020 | India | 50 | Female | B.1.217 | First |
| hCoV-19/India/MH-ACTREC-638/2020 | betacoronavirus | EPI_ISL_700271 | 13-09-2020 | India | 28 | Female | B.1.210 | First |
| hCoV-19/India/MH-ACTREC-639/2020 | betacoronavirus | EPI_ISL_700272 | 13-09-2020 | India | 72 | Female | B.1.1.286 | First |
| hCoV-19/India/MH-ACTREC-640/2020 | betacoronavirus | EPI_ISL_700273 | 14-09-2020 | India | 47 | Male | B.1.36.6 | First |
| hCoV-19/India/MH-ACTREC-641/2020 | betacoronavirus | EPI_ISL_700274 | 14-09-2020 | India | 26 | Female | B.1.36 | First |
| hCoV-19/India/MH-ACTREC-642/2020 | betacoronavirus | EPI_ISL_700275 | 14-09-2020 | India | 47 | Male | B.1.36.1 | First |
| hCoV-19/India/MH-ACTREC-643/2020 | betacoronavirus | EPI_ISL_723069 | 14-09-2020 | India | 50 | Female | B.1.36 | First |
| hCoV-19/India/MH-ACTREC-644/2020 | betacoronavirus | EPI_ISL_723070 | 14-09-2020 | India | 42 | Male | B.1.1.306 | First |
| hCoV-19/India/MH-ACTREC-645/2020 | betacoronavirus | EPI_ISL_723071 | 14-09-2020 | India | 41 | Female | B.1.1.306 | First |
| hCoV-19/India/MH-ACTREC-646/2020 | betacoronavirus | EPI_ISL_700276 | 14-09-2020 | India | 29 | Male | B.1.36 | First |
| hCoV-19/India/MH-ACTREC-647/2020 | betacoronavirus | EPI_ISL_700277 | 14-09-2020 | India | 28 | Male | B.1.36 | First |
| hCoV-19/India/MH-ACTREC-648/2020 | betacoronavirus | EPI_ISL_700278 | 14-09-2020 | India | 21 | Male | B.1.1.281 | First |
| hCoV-19/India/MH-ACTREC-649/2020 | betacoronavirus | EPI_ISL_700279 | 14-09-2020 | India | 19 | Male | B.1.1.281 | First |
| hCoV-19/India/MH-ACTREC-650/2020 | betacoronavirus | EPI_ISL_700280 | 14-09-2020 | India | 38 | Male | B.1.1.306 | First |
| hCoV-19/India/MH-ACTREC-651/2020 | betacoronavirus | EPI_ISL_700281 | 14-09-2020 | India | 50 | Female | B.1.1.212 | First |
| hCoV-19/India/MH-ACTREC-652/2020 | betacoronavirus | EPI_ISL_700282 | 14-09-2020 | India | 48 | Male | B.1.1.281 | First |
| hCoV-19/India/MH-ACTREC-653/2020 | betacoronavirus | EPI_ISL_700283 | 14-09-2020 | India | 36 | Female | B.1.1.281 | First |
| hCoV-19/India/MH-ACTREC-654/2020 | betacoronavirus | EPI_ISL_700284 | 14-09-2020 | India | 17 | Female | B.1.1.281 | First |
| hCoV-19/India/MH-ACTREC-655/2020 | betacoronavirus | EPI_ISL_700285 | 14-09-2020 | India | 8 | Male | B.1.1.281 | First |
| hCoV-19/India/MH-ACTREC-656/2020 | betacoronavirus | EPI_ISL_700286 | 14-09-2020 | India | 57 | Male | B.1.1.281 | First |
| hCoV-19/India/MH-ACTREC-657/2020 | betacoronavirus | EPI_ISL_700287 | 14-09-2020 | India | 80 | Female | B.1.1.281 | First |
| hCoV-19/India/MH-ACTREC-658/2020 | betacoronavirus | EPI_ISL_700288 | 15-09-2020 | India | 73 | Male | B.1.210 | First |
| hCoV-19/India/MH-ACTREC-659/2020 | betacoronavirus | EPI_ISL_700289 | 15-09-2020 | India | 25 | Male | B.1.36 | First |
| hCoV-19/India/MH-ACTREC-660/2020 | betacoronavirus | EPI_ISL_700290 | 15-09-2020 | India | 56 | Male | B.1.1.306 | First |
| hCoV-19/India/MH-ACTREC-661/2020 | betacoronavirus | EPI_ISL_723072 | 15-09-2020 | India | 49 | Female | B.1.1.306 | First |
| hCoV-19/India/MH-ACTREC-662/2020 | betacoronavirus | EPI_ISL_700291 | 15-09-2020 | India | 41 | Female | B.1.1.306 | First |
| hCoV-19/India/MH-ACTREC-663/2020 | betacoronavirus | EPI_ISL_700292 | 16-09-2020 | India | 27 | Male | B.1.210 | First |
| hCoV-19/India/MH-ACTREC-664/2020 | betacoronavirus | EPI_ISL_700293 | 16-09-2020 | India | 35 | Female | B.1.36.1 | First |
| hCoV-19/India/MH-ACTREC-665/2020 | betacoronavirus | EPI_ISL_723073 | 16-09-2020 | India | 27 | Male | B.1.178 | First |
| hCoV-19/India/MH-ACTREC-666/2020 | betacoronavirus | EPI_ISL_723074 | 16-09-2020 | India | 33 | Male | B.1.1.306 | First |
| hCoV-19/India/MH-ACTREC-667/2020 | betacoronavirus | EPI_ISL_723075 | 16-09-2020 | India | 37 | Male | B.1.1.306 | First |
| hCoV-19/India/MH-ACTREC-668/2020 | betacoronavirus | EPI_ISL_700294 | 16-09-2020 | India | 27 | Male | B.1.1.216 | First |
| hCoV-19/India/MH-ACTREC-669/2020 | betacoronavirus | EPI_ISL_700295 | 16-09-2020 | India | 42 | Male | B.1.1.306 | First |
| hCoV-19/India/MH-ACTREC-670/2020 | betacoronavirus | EPI_ISL_700296 | 18-09-2020 | India | 46 | Male | B.1.1.306 | First |
| hCoV-19/India/MH-ACTREC-671/2020 | betacoronavirus | EPI_ISL_700297 | 18-09-2020 | India | 26 | Female | B.1.1.306 | First |
| hCoV-19/India/MH-ACTREC-672/2020 | betacoronavirus | EPI_ISL_700298 | 18-09-2020 | India | 42 | Male | B.1.1.306 | First |
| hCoV-19/India/MH-ACTREC-673/2020 | betacoronavirus | EPI_ISL_700299 | 18-09-2020 | India | 30 | Male | B.1.36 | First |
| hCoV-19/India/MH-ACTREC-674/2020 | betacoronavirus | EPI_ISL_723076 | 18-09-2020 | India | 33 | Female | B.1.1.306 | First |
| hCoV-19/India/MH-ACTREC-675/2020 | betacoronavirus | EPI_ISL_700300 | 18-09-2020 | India | 45 | Male | B.1.1.281 | First |
| hCoV-19/India/MH-ACTREC-676/2020 | betacoronavirus | EPI_ISL_700301 | 18-09-2020 | India | 49 | Male | B.1.1.212 | First |
| hCoV-19/India/MH-ACTREC-677/2020 | betacoronavirus | EPI_ISL_700302 | 18-09-2020 | India | 70 | Male | B.1.1.306 | First |
| hCoV-19/India/MH-ACTREC-678/2020 | betacoronavirus | EPI_ISL_700303 | 18-09-2020 | India | 40 | Male | B.1.1.281 | First |
| hCoV-19/India/MH-ACTREC-679/2020 | betacoronavirus | EPI_ISL_700304 | 19-09-2020 | India | 69 | Male | B.1.1.212 | First |
| hCoV-19/India/MH-ACTREC-680/2020 | betacoronavirus | EPI_ISL_700305 | 19-09-2020 | India | 40 | Male | B.1.19 | First |
| hCoV-19/India/UP-ACTREC-681/2020 | betacoronavirus | EPI_ISL_700306 | 19-09-2020 | India | 23 | Male | B.1.36 | First |
| hCoV-19/India/MH-ACTREC-682/2020 | betacoronavirus | EPI_ISL_700307 | 19-09-2020 | India | 12 | Female | B.1.1.306 | First |
| hCoV-19/India/MH-ACTREC-683/2020 | betacoronavirus | EPI_ISL_700308 | 20-09-2020 | India | 52 | Female | B.1.1.281 | First |
| hCoV-19/India/MH-ACTREC-684/2020 | betacoronavirus | EPI_ISL_700309 | 22-09-2020 | India | 47 | Female | B.1.1.306 | First |
| hCoV-19/India/MH-ACTREC-685/2020 | betacoronavirus | EPI_ISL_700310 | 22-09-2020 | India | 17 | Female | B.1.1.281 | First |
| hCoV-19/India/WB-ACTREC-686/2020 | betacoronavirus | EPI_ISL_700311 | 23-09-2020 | India | 34 | Male | B.1.1.216 | First |
| hCoV-19/India/MH-ACTREC-687/2020 | betacoronavirus | EPI_ISL_700312 | 23-09-2020 | India | 61 | Female | B.1.1.306 | First |
| hCoV-19/India/OR-ACTREC-688/2020 | betacoronavirus | EPI_ISL_700313 | 24-09-2020 | India | 43 | Female | B.1.1 | First |
| hCoV-19/India/MH-ACTREC-689/2020 | betacoronavirus | EPI_ISL_700314 | 24-09-2020 | India | 35 | Male | B.1.1 | First |
| hCoV-19/India/MH-ACTREC-690/2020 | betacoronavirus | EPI_ISL_700315 | 24-09-2020 | India | 34 | Male | B.1.1.281 | First |
| hCoV-19/India/MH-ACTREC-691/2020 | betacoronavirus | EPI_ISL_700316 | 24-09-2020 | India | 64 | Male | B.1.1.281 | First |
| hCoV-19/India/MH-ACTREC-692/2020 | betacoronavirus | EPI_ISL_700317 | 24-09-2020 | India | 28 | Female | B.1.1.306 | First |
| hCoV-19/India/MH-ACTREC-693/2020 | betacoronavirus | EPI_ISL_700318 | 24-09-2020 | India | 31 | Male | B.1.1.281 | First |
| hCoV-19/India/MH-ACTREC-694/2020 | betacoronavirus | EPI_ISL_700319 | 25-09-2020 | India | 37 | Female | B.1.1.216 | First |
| hCoV-19/India/MH-ACTREC-695/2020 | betacoronavirus | EPI_ISL_700320 | 25-09-2020 | India | 48 | Male | B.1 | First |
| hCoV-19/India/MH-ACTREC-696/2020 | betacoronavirus | EPI_ISL_700321 | 25-09-2020 | India | 65 | Female | B.1.210 | First |
| hCoV-19/India/MH-ACTREC-697/2020 | betacoronavirus | EPI_ISL_700322 | 25-09-2020 | India | 51 | Male | B.1.36.16 | First |
| hCoV-19/India/MH-ACTREC-698/2020 | betacoronavirus | EPI_ISL_700323 | 25-09-2020 | India | 30 | Male | B.1.217 | First |
| hCoV-19/India/MH-ACTREC-699/2020 | betacoronavirus | EPI_ISL_700324 | 25-09-2020 | India | 23 | Female | B.1.1.306 | First |
| hCoV-19/India/MH-ACTREC-700/2020 | betacoronavirus | EPI_ISL_700325 | 25-09-2020 | India | 20 | Male | B.1.1.306 | First |
| hCoV-19/India/MH-ACTREC-001/2021 | betacoronavirus | EPI_ISL_2530111 | 01-04-2021 | India | 30 | Male | B.1.617.1 | Second |
| hCoV-19/India/MH-ACTREC-002/2021 | betacoronavirus | EPI_ISL_2530113 | 03-04-2021 | India | 69 | Male | B.1.617.1 | Second |
| hCoV-19/India/MH-ACTREC-003/2021 | betacoronavirus | EPI_ISL_2530115 | 03-04-2021 | India | 20 | Female | B.1.617.2 | Second |
| hCoV-19/India/MH-ACTREC-004/2021 | betacoronavirus | EPI_ISL_2530117 | 03-04-2021 | India | 51 | Male | B.1.617.2 | Second |
| hCoV-19/India/MH-ACTREC-005/2021 | betacoronavirus | EPI_ISL_2530119 | 03-04-2021 | India | 69 | Male | B.1.617.2 | Second |
| hCoV-19/India/MH-ACTREC-006/2021 | betacoronavirus | EPI_ISL_2530120 | 03-04-2021 | India | 33 | Female | B.1.617.1 | Second |
| hCoV-19/India/MH-ACTREC-007/2021 | betacoronavirus | EPI_ISL_2530122 | 03-04-2021 | India | 25 | Female | B.1.617.1 | Second |
| hCoV-19/India/MH-ACTREC-008/2021 | betacoronavirus | EPI_ISL_2530124 | 03-04-2021 | India | 63 | Female | B.1.617.1 | Second |
| hCoV-19/India/MH-ACTREC-009/2021 | betacoronavirus | EPI_ISL_2600953 | 03-04-2021 | India | 58 | Male | B.1.617.1 | Second |
| hCoV-19/India/MH-ACTREC-010/2021 | betacoronavirus | EPI_ISL_2530126 | 03-04-2021 | India | 33 | Female | B.1.617.1 | Second |
| hCoV-19/India/MH-ACTREC-011/2021 | betacoronavirus | EPI_ISL_2530128 | 03-04-2021 | India | 43 | Male | B.1.617.1 | Second |
| hCoV-19/India/MH-ACTREC-012/2021 | betacoronavirus | EPI_ISL_2530129 | 03-04-2021 | India | 32 | Female | B.1.617.1 | Second |
| hCoV-19/India/MH-ACTREC-013/2021 | betacoronavirus | EPI_ISL_2530131 | 05-04-2021 | India | 22 | Male | B.1.617.1 | Second |
| hCoV-19/India/MH-ACTREC-014/2021 | betacoronavirus | EPI_ISL_2530132 | 05-04-2021 | India | 30 | Male | B.1.617.2 | Second |
| hCoV-19/India/MH-ACTREC-015/2021 | betacoronavirus | EPI_ISL_2530134 | 05-04-2021 | India | 37 | Female | B.1.617.2 | Second |
| hCoV-19/India/MH-ACTREC-016/2021 | betacoronavirus | EPI_ISL_2530136 | 05-04-2021 | India | 40 | Male | B.1.617.1 | Second |
| hCoV-19/India/MH-ACTREC-017/2021 | betacoronavirus | EPI_ISL_2530137 | 05-04-2021 | India | 73 | Male | B.1.617.1 | Second |
| hCoV-19/India/MH-ACTREC-018/2021 | betacoronavirus | EPI_ISL_2530139 | 05-04-2021 | India | 61 | Female | B.1.617.1 | Second |
| hCoV-19/India/MH-ACTREC-019/2021 | betacoronavirus | EPI_ISL_2600954 | 05-04-2021 | India | 23 | Female | B.1.617.1 | Second |
| hCoV-19/India/MH-ACTREC-020/2021 | betacoronavirus | EPI_ISL_2600955 | 05-04-2021 | India | 53 | Female | B.1.617.1 | Second |
| hCoV-19/India/MH-ACTREC-021/2021 | betacoronavirus | EPI_ISL_2530141 | 05-04-2021 | India | 34 | Female | B.1.617.1 | Second |
| hCoV-19/India/MH-ACTREC-022/2021 | betacoronavirus | EPI_ISL_2600956 | 05-04-2021 | India | 27 | Female | B.1.617.1 | Second |
| hCoV-19/India/MH-ACTREC-023/2021 | betacoronavirus | EPI_ISL_2600957 | 05-04-2021 | India | 29 | Male | B.1.617.1 | Second |
| hCoV-19/India/MH-ACTREC-024/2021 | betacoronavirus | EPI_ISL_2530143 | 05-04-2021 | India | 32 | Male | B.1.617.2 | Second |
| hCoV-19/India/MH-ACTREC-025/2021 | betacoronavirus | EPI_ISL_2530144 | 05-04-2021 | India | 33 | Female | B.1.617.1 | Second |
| hCoV-19/India/MH-ACTREC-026/2021 | betacoronavirus | EPI_ISL_2530146 | 06-04-2021 | India | 61 | Female | B.1.617.1 | Second |
| hCoV-19/India/MH-ACTREC-027/2021 | betacoronavirus | EPI_ISL_2530148 | 06-04-2021 | India | 33 | Female | B.1.617.2 | Second |
| hCoV-19/India/MH-ACTREC-028/2021 | betacoronavirus | EPI_ISL_2530149 | 06-04-2021 | India | 23 | Female | B.1.617.1 | Second |
| hCoV-19/India/MH-ACTREC-029/2021 | betacoronavirus | EPI_ISL_2530151 | 06-04-2021 | India | 29 | Male | B.1.617.1 | Second |
| hCoV-19/India/MH-ACTREC-030/2021 | betacoronavirus | EPI_ISL_2530152 | 10-04-2021 | India | 23 | Male | B.1.1.7 | Second |
| hCoV-19/India/MH-ACTREC-031/2021 | betacoronavirus | EPI_ISL_2530154 | 07-04-2021 | India | 59 | Female | B.1.617.1 | Second |
| hCoV-19/India/MH-ACTREC-032/2021 | betacoronavirus | EPI_ISL_2600958 | 07-04-2021 | India | 29 | Female | B.1.617.2 | Second |
| hCoV-19/India/MH-ACTREC-033/2021 | betacoronavirus | EPI_ISL_2530155 | 07-04-2021 | India | 42 | Female | B.1.617.1 | Second |
| hCoV-19/India/MH-ACTREC-034/2021 | betacoronavirus | EPI_ISL_2530157 | 07-04-2021 | India | 23 | Male | B.1.617.1 | Second |
| hCoV-19/India/MH-ACTREC-035/2021 | betacoronavirus | EPI_ISL_2600959 | 07-04-2021 | India | 69 | Male | B.1.617.3 | Second |
| hCoV-19/India/MH-ACTREC-036/2021 | betacoronavirus | EPI_ISL_2530158 | 08-04-2021 | India | 38 | Female | B.1.617.1 | Second |
| hCoV-19/India/MH-ACTREC-037/2021 | betacoronavirus | EPI_ISL_2530160 | 08-04-2021 | India | 32 | Male | B.1.617.2 | Second |
| hCoV-19/India/MH-ACTREC-038/2021 | betacoronavirus | EPI_ISL_2530161 | 08-04-2021 | India | 25 | Female | B.1.617.2 | Second |
| hCoV-19/India/MH-ACTREC-039/2021 | betacoronavirus | EPI_ISL_2530163 | 08-04-2021 | India | 30 | Male | B.1.617.2 | Second |
| hCoV-19/India/MH-ACTREC-040/2021 | betacoronavirus | EPI_ISL_2530165 | 08-04-2021 | India | 27 | Female | B.1.617.1 | Second |
| hCoV-19/India/MH-ACTREC-041/2021 | betacoronavirus | EPI_ISL_2530167 | 09-04-2021 | India | 40 | Male | B.1.617.1 | Second |
| hCoV-19/India/MH-ACTREC-042/2021 | betacoronavirus | EPI_ISL_2530169 | 09-04-2021 | India | 28 | Male | B.1.617.2 | Second |
| hCoV-19/India/MH-ACTREC-043/2021 | betacoronavirus | EPI_ISL_2530170 | 09-04-2021 | India | 39 | Female | B.1.617.1 | Second |
| hCoV-19/India/MH-ACTREC-044/2021 | betacoronavirus | EPI_ISL_2530172 | 09-04-2021 | India | 28 | Male | B.1.617.1 | Second |
| hCoV-19/India/MH-ACTREC-045/2021 | betacoronavirus | EPI_ISL_2530174 | 09-04-2021 | India | 24 | Female | B.1.617.2 | Second |
| hCoV-19/India/MH-ACTREC-046/2021 | betacoronavirus | EPI_ISL_2530176 | 09-04-2021 | India | 26 | Female | B.1.617.1 | Second |
| hCoV-19/India/MH-ACTREC-047/2021 | betacoronavirus | EPI_ISL_2530177 | 10-04-2021 | India | 38 | Female | B.1.617.1 | Second |
| hCoV-19/India/MH-ACTREC-048/2021 | betacoronavirus | EPI_ISL_2530179 | 10-04-2021 | India | 20 | Male | B.1.617.2 | Second |
| hCoV-19/India/MH-ACTREC-049/2021 | betacoronavirus | EPI_ISL_2530180 | 10-04-2021 | India | 32 | Female | B.1.617.1 | Second |
| hCoV-19/India/MH-ACTREC-050/2021 | betacoronavirus | EPI_ISL_2530182 | 10-04-2021 | India | 23 | Female | B.1.617.1 | Second |
| hCoV-19/India/MH-ACTREC-051/2021 | betacoronavirus | EPI_ISL_2530183 | 10-04-2021 | India | 42 | Male | B.1.617.1 | Second |
| hCoV-19/India/MH-ACTREC-052/2021 | betacoronavirus | EPI_ISL_2530185 | 11-04-2021 | India | 48 | Male | B.1.617.2 | Second |
| hCoV-19/India/MH-ACTREC-053/2021 | betacoronavirus | EPI_ISL_2530186 | 12-04-2021 | India | 29 | Male | B.1.617.2 | Second |
| hCoV-19/India/MH-ACTREC-054/2021 | betacoronavirus | EPI_ISL_2530187 | 12-04-2021 | India | 39 | Male | B.1.617.1 | Second |
| hCoV-19/India/MH-ACTREC-055/2021 | betacoronavirus | EPI_ISL_2530188 | 12-04-2021 | India | 38 | Male | B.1.617.1 | Second |
| hCoV-19/India/MH-ACTREC-056/2021 | betacoronavirus | EPI_ISL_2530190 | 12-04-2021 | India | 28 | Female | B.1.617.1 | Second |
| hCoV-19/India/MH-ACTREC-057/2021 | betacoronavirus | EPI_ISL_2530191 | 12-04-2021 | India | 35 | Female | B.1.617.2 | Second |
| hCoV-19/India/MH-ACTREC-058/2021 | betacoronavirus | EPI_ISL_2530193 | 12-04-2021 | India | 26 | Female | B.1.617.2 | Second |
| hCoV-19/India/MH-ACTREC-059/2021 | betacoronavirus | EPI_ISL_2530195 | 12-04-2021 | India | 40 | Male | B.1.617.2 | Second |
| hCoV-19/India/MH-ACTREC-060/2021 | betacoronavirus | EPI_ISL_2530196 | 13-04-2021 | India | 58 | Female | B.1.617.2 | Second |
| hCoV-19/India/MH-ACTREC-061/2021 | betacoronavirus | EPI_ISL_2530198 | 13-04-2021 | India | 28 | Male | B.1.617.2 | Second |
| hCoV-19/India/MH-ACTREC-062/2021 | betacoronavirus | EPI_ISL_2530199 | 13-04-2021 | India | 32 | Male | B.1.617.2 | Second |
| hCoV-19/India/MH-ACTREC-063/2021 | betacoronavirus | EPI_ISL_2530201 | 13-04-2021 | India | 56 | Male | B.1.617.2 | Second |
| hCoV-19/India/MH-ACTREC-064/2021 | betacoronavirus | EPI_ISL_2530202 | 14-04-2021 | India | 28 | Male | B.1.617.2 | Second |
| hCoV-19/India/MH-ACTREC-065/2021 | betacoronavirus | EPI_ISL_2600960 | 14-04-2021 | India | 31 | Male | B.1.617.1 | Second |
| hCoV-19/India/MH-ACTREC-066/2021 | betacoronavirus | EPI_ISL_2530204 | 15-04-2021 | India | 55 | Male | B.1.617.2 | Second |
| hCoV-19/India/MH-ACTREC-067/2021 | betacoronavirus | EPI_ISL_2530206 | 16-04-2021 | India | 30 | Male | B.1.617.2 | Second |
| hCoV-19/India/MH-ACTREC-068/2021 | betacoronavirus | EPI_ISL_2600961 | 16-04-2021 | India | 58 | Female | B.1.617.3 | Second |
| hCoV-19/India/MH-ACTREC-069/2021 | betacoronavirus | EPI_ISL_2530207 | 17-04-2021 | India | 54 | Female | B.1.617.2 | Second |
| hCoV-19/India/MH-ACTREC-070/2021 | betacoronavirus | EPI_ISL_2530209 | 17-04-2021 | India | 37 | Female | B.1.617.2 | Second |
| hCoV-19/India/MH-ACTREC-071/2021 | betacoronavirus | EPI_ISL_2530210 | 17-04-2021 | India | 40 | Female | B.1.617.2 | Second |
| hCoV-19/India/MH-ACTREC-072/2021 | betacoronavirus | EPI_ISL_2530212 | 18-04-2021 | India | 30 | Male | B.1.617.2 | Second |
| hCoV-19/India/MH-ACTREC-073/2021 | betacoronavirus | EPI_ISL_2530214 | 19-04-2021 | India | 29 | Female | B.1.617.2 | Second |
| hCoV-19/India/MH-ACTREC-074/2021 | betacoronavirus | EPI_ISL_2530216 | 19-04-2021 | India | 26 | Female | B.1.617.1 | Second |
| hCoV-19/India/MH-ACTREC-075/2021 | betacoronavirus | EPI_ISL_2530217 | 20-04-2021 | India | 49 | Female | B.1.617.2 | Second |
| hCoV-19/India/MH-ACTREC-076/2021 | betacoronavirus | EPI_ISL_2530219 | 20-04-2021 | India | 71 | Female | B.1.617.2 | Second |
| hCoV-19/India/MH-ACTREC-077/2021 | betacoronavirus | EPI_ISL_2530221 | 20-04-2021 | India | 41 | Male | B.1.617.2 | Second |
| hCoV-19/India/MH-ACTREC-078/2021 | betacoronavirus | EPI_ISL_2530222 | 22-04-2021 | India | 49 | Male | B.1.617.2 | Second |
| hCoV-19/India/MH-ACTREC-079/2021 | betacoronavirus | EPI_ISL_2530224 | 22-04-2021 | India | 31 | Female | B.1.617.2 | Second |
| hCoV-19/India/MH-ACTREC-080/2021 | betacoronavirus | EPI_ISL_2530226 | 23-04-2021 | India | 47 | Male | B.1.617.2 | Second |
| hCoV-19/India/MH-ACTREC-081/2021 | betacoronavirus | EPI_ISL_2530227 | 23-04-2021 | India | 23 | Male | B.1.617.2 | Second |
| hCoV-19/India/MH-ACTREC-082/2021 | betacoronavirus | EPI_ISL_2530229 | 23-04-2021 | India | 45 | Female | B.1.617.2 | Second |
| hCoV-19/India/MH-ACTREC-083/2021 | betacoronavirus | EPI_ISL_2530230 | 23-04-2021 | India | 25 | Female | B.1.617.2 | Second |
| hCoV-19/India/MH-ACTREC-084/2021 | betacoronavirus | EPI_ISL_2530232 | 24-04-2021 | India | 39 | Male | B.1.617.2 | Second |
| hCoV-19/India/MH-ACTREC-085/2021 | betacoronavirus | EPI_ISL_2530233 | 26-04-2021 | India | 48 | Female | B.1 | Second |
| hCoV-19/India/MH-ACTREC-086/2021 | betacoronavirus | EPI_ISL_2530235 | 26-04-2021 | India | 52 | Male | B.1 | Second |
| hCoV-19/India/MH-ACTREC-087/2021 | betacoronavirus | EPI_ISL_2530236 | 26-04-2021 | India | 25 | Female | B.1.617.2 | Second |
| hCoV-19/India/MH-ACTREC-088/2021 | betacoronavirus | EPI_ISL_2530238 | 26-04-2021 | India | 39 | Male | B.1.617.1 | Second |
| hCoV-19/India/MH-ACTREC-089/2021 | betacoronavirus | EPI_ISL_2530239 | 27-04-2021 | India | 27 | Female | B.1.617.2 | Second |
| hCoV-19/India/MH-ACTREC-090/2021 | betacoronavirus | EPI_ISL_2530241 | 28-04-2021 | India | 55 | Male | B.1.617.2 | Second |
| hCoV-19/India/MH-ACTREC-091/2021 | betacoronavirus | EPI_ISL_2530243 | 28-04-2021 | India | 31 | Male | B.1.617.2 | Second |
| hCoV-19/India/MH-ACTREC-092/2021 | betacoronavirus | EPI_ISL_2530245 | 29-04-2021 | India | 55 | Female | B.1.617.2 | Second |
| hCoV-19/India/MH-ACTREC-093/2021 | betacoronavirus | EPI_ISL_2530246 | 29-04-2021 | India | 64 | Male | B.1.617.2 | Second |
| hCoV-19/India/MH-ACTREC-094/2021 | betacoronavirus | EPI_ISL_2530248 | 19-04-2021 | India | 31 | Female | B.1.617.2 | Second |
| hCoV-19/India/MH-ACTREC-095/2021 | betacoronavirus | EPI_ISL_2530250 | 03-05-2021 | India | 27 | Female | B.1.617.2 | Second |
| hCoV-19/India/MH-ACTREC-096/2021 | betacoronavirus | EPI_ISL_2530252 | 03-05-2021 | India | 43 | Female | B.1.617.2 | Second |
| hCoV-19/India/MH-ACTREC-097/2021 | betacoronavirus | EPI_ISL_2530253 | 04-05-2021 | India | 39 | Male | B.1.617.2 | Second |
| hCoV-19/India/MH-ACTREC-098/2021 | betacoronavirus | EPI_ISL_2530255 | 04-05-2021 | India | 52 | Male | B.1.617.2 | Second |
| hCoV-19/India/MH-ACTREC-099/2021 | betacoronavirus | EPI_ISL_2530256 | 10-05-2021 | India | 55 | Female | B.1.617.2 | Second |
| hCoV-19/India/MH-ACTREC-100/2021 | betacoronavirus | EPI_ISL_2530258 | 10-05-2021 | India | 57 | Male | B.1.617.2 | Second |
| hCoV-19/India/MH-ACTREC-101/2021 | betacoronavirus | EPI_ISL_2530259 | 10-05-2021 | India | 20 | Female | B.1.617.2 | Second |
| hCoV-19/India/MH-ACTREC-102/2021 | betacoronavirus | EPI_ISL_2530261 | 12-05-2021 | India | 23 | Female | B.1.617.1 | Second |
| hCoV-19/India/MH-ACTREC-103/2021 | betacoronavirus | EPI_ISL_2530262 | 13-05-2021 | India | 39 | Female | B.1.617.2 | Second |
| hCoV-19/India/MH-ACTREC-104/2021 | betacoronavirus | EPI_ISL_2530264 | 15-05-2021 | India | 59 | Male | B.1.617.2 | Second |
| hCoV-19/India/MH-ACTREC-105/2021 | betacoronavirus | EPI_ISL_2530265 | 06-04-2021 | India | 46 | Female | B.1.617.2 | Second |
| hCoV-19/India/MH-ACTREC-106/2021 | betacoronavirus | EPI_ISL_2530267 | 20-04-2021 | India | 35 | Male | B.1.617.1 | Second |
| hCoV-19/India/MH-ACTREC-107/2021 | betacoronavirus | EPI_ISL_2530268 | 03-05-2021 | India | 29 | Male | B.1.617.2 | Second |
| hCoV-19/India/MH-ACTREC-108/2021 | betacoronavirus | EPI_ISL_2530270 | 05-05-2021 | India | 45 | Male | B.1.617.2 | Second |
| hCoV-19/India/MH-ACTREC-109/2021 | betacoronavirus | EPI_ISL_2530272 | 07-04-2021 | India | 29 | Female | B.1.617.1 | Second |
| hCoV-19/India/MH-ACTREC-110/2021 | betacoronavirus | EPI_ISL_2530273 | 07-04-2021 | India | 29 | Female | B.1.617.1 | Second |
| hCoV-19/India/MH-ACTREC-111/2021 | betacoronavirus | EPI_ISL_2530275 | 07-04-2021 | India | 22 | Male | B.1.617.1 | Second |
| hCoV-19/India/MH-ACTREC-112/2021 | betacoronavirus | EPI_ISL_2530276 | 08-04-2021 | India | 44 | Male | B.1.617.1 | Second |
| hCoV-19/India/MH-ACTREC-113/2021 | betacoronavirus | EPI_ISL_2530278 | 10-04-2021 | India | 48 | Female | B.1.617.1 | Second |
| hCoV-19/India/MH-ACTREC-114/2021 | betacoronavirus | EPI_ISL_2530279 | 13-04-2021 | India | 55 | Male | B.1.617.1 | Second |
| hCoV-19/India/MH-ACTREC-115/2021 | betacoronavirus | EPI_ISL_2530281 | 13-04-2021 | India | 31 | Female | B.1.617.1 | Second |
| hCoV-19/India/MH-ACTREC-116/2021 | betacoronavirus | EPI_ISL_2530283 | 17-04-2021 | India | 53 | Male | B.1.617.1 | Second |
| hCoV-19/India/MH-ACTREC-117/2021 | betacoronavirus | EPI_ISL_2530285 | 22-04-2021 | India | 50 | Male | B.1.617.1 | Second |
| hCoV-19/India/MH-ACTREC-118/2021 | betacoronavirus | EPI_ISL_2530286 | 03-05-2021 | India | 66 | Male | B.1.617.1 | Second |
| hCoV-19/India/MH-ACTREC-119/2021 | betacoronavirus | EPI_ISL_2530290 | 10-05-2021 | India | 77 | Male | B.1.617.2 | Second |
| hCoV-19/India/MH-ACTREC-120/2021 | betacoronavirus | EPI_ISL_2530295 | 29-03-2021 | India | 33 | Female | B.1.617.1 | Second |
| hCoV-19/India/MH-ACTREC-121/2021 | betacoronavirus | EPI_ISL_2600962 | 05-04-2021 | India | 24 | Male | B.1.617.1 | Second |
| hCoV-19/India/MH-ACTREC-122/2021 | betacoronavirus | EPI_ISL_2530299 | 12-04-2021 | India | 15 | Female | B.1.617.2 | Second |
| hCoV-19/India/MH-ACTREC-123/2021 | betacoronavirus | EPI_ISL_2530304 | 12-04-2021 | India | 48 | Female | B.1.617.2 | Second |
| hCoV-19/India/MH-ACTREC-124/2021 | betacoronavirus | EPI_ISL_2530308 | 12-04-2021 | India | 25 | Female | B.1.617.2 | Second |
| hCoV-19/India/MH-ACTREC-125/2021 | betacoronavirus | EPI_ISL_2530313 | 17-03-2021 | India | 31 | Female | B.1.617.1 | Second |
| hCoV-19/India/MH-ACTREC-126/2021 | betacoronavirus | EPI_ISL_2530317 | 19-03-2021 | India | 56 | Male | B.1.617.1 | Second |
| hCoV-19/India/MH-ACTREC-127/2021 | betacoronavirus | EPI_ISL_2530319 | 20-03-2021 | India | 29 | Female | B.1.617.2 | Second |
| hCoV-19/India/MH-ACTREC-128/2021 | betacoronavirus | EPI_ISL_2530321 | 20-03-2021 | India | 45 | Female | B.1.617.1 | Second |
| hCoV-19/India/MH-ACTREC-129/2021 | betacoronavirus | EPI_ISL_2530322 | 23-03-2021 | India | 62 | Female | B.1.36.22 | Second |
| hCoV-19/India/MH-ACTREC-130/2021 | betacoronavirus | EPI_ISL_2530324 | 23-03-2021 | India | 35 | Female | B.1.617.1 | Second |
| hCoV-19/India/MH-ACTREC-131/2021 | betacoronavirus | EPI_ISL_2530326 | 23-03-2021 | India | 26 | Male | B.1.617.1 | Second |
| hCoV-19/India/MH-ACTREC-132/2021 | betacoronavirus | EPI_ISL_2530330 | 23-03-2021 | India | 29 | Male | B.1.617.1 | Second |
| hCoV-19/India/MH-ACTREC-133/2021 | betacoronavirus | EPI_ISL_2600963 | 23-03-2021 | India | 66 | Male | B.1.617.3 | Second |
| hCoV-19/India/MH-ACTREC-134/2021 | betacoronavirus | EPI_ISL_2530334 | 24-03-2021 | India | 29 | Female | B.1.617.1 | Second |
| hCoV-19/India/MH-ACTREC-135/2021 | betacoronavirus | EPI_ISL_2530336 | 25-03-2021 | India | 52 | Male | B.1.617.1 | Second |
| hCoV-19/India/MH-ACTREC-136/2021 | betacoronavirus | EPI_ISL_2530337 | 26-03-2021 | India | 71 | Male | B.1.617.1 | Second |
| hCoV-19/India/MH-ACTREC-137/2021 | betacoronavirus | EPI_ISL_2530339 | 26-03-2021 | India | 40 | Male | B.1.617.1 | Second |
| hCoV-19/India/MH-ACTREC-138/2021 | betacoronavirus | EPI_ISL_2530340 | 26-03-2021 | India | 32 | Male | B.1.617.1 | Second |
| hCoV-19/India/MH-ACTREC-139/2021 | betacoronavirus | EPI_ISL_2530344 | 27-03-2021 | India | 49 | Female | B.1.617.1 | Second |
| hCoV-19/India/MH-ACTREC-140/2021 | betacoronavirus | EPI_ISL_2530349 | 27-03-2021 | India | 34 | Female | B.1.617.1 | Second |
| hCoV-19/India/MH-ACTREC-141/2021 | betacoronavirus | EPI_ISL_2530353 | 27-03-2021 | India | 6 | Male | B.1.617.1 | Second |
| hCoV-19/India/MH-ACTREC-142/2021 | betacoronavirus | EPI_ISL_2530357 | 27-03-2021 | India | 30 | Female | B.1.617.1 | Second |
| hCoV-19/India/MH-ACTREC-143/2021 | betacoronavirus | EPI_ISL_2530360 | 30-03-2021 | India | 49 | Male | B.1.617.1 | Second |
| hCoV-19/India/MH-ACTREC-144/2021 | betacoronavirus | EPI_ISL_2530364 | 30-03-2021 | India | 36 | Male | B.1.617.1 | Second |
| hCoV-19/India/MH-ACTREC-145/2021 | betacoronavirus | EPI_ISL_2530366 | 30-03-2021 | India | 31 | Male | B.1.617.1 | Second |
| hCoV-19/India/MH-ACTREC-146/2021 | betacoronavirus | EPI_ISL_2530367 | 30-03-2021 | India | 39 | Female | B.1.617.1 | Second |
| hCoV-19/India/MH-ACTREC-147/2021 | betacoronavirus | EPI_ISL_2530369 | 31-03-2021 | India | 42 | Male | B.1.617.1 | Second |
| hCoV-19/India/MH-ACTREC-148/2021 | betacoronavirus | EPI_ISL_2530371 | 31-03-2021 | India | 26 | Female | B.1.617.1 | Second |
| hCoV-19/India/MH-ACTREC-149/2021 | betacoronavirus | EPI_ISL_2530373 | 18-04-2021 | India | 30 | Male | B.1.617.2 | Second |
| hCoV-19/India/MH-ACTREC-150/2021 | betacoronavirus | EPI_ISL_2530374 | 29-04-2021 | India | 40 | Female | B.1.617.2 | Second |
| hCoV-19/India/MH-ACTREC-151/2021 | betacoronavirus | EPI_ISL_2530376 | 15-04-2021 | India | 61 | Male | B.1.617.1 | Second |
| hCoV-19/India/MH-ACTREC-152/2021 | betacoronavirus | EPI_ISL_2530378 | 28-04-2021 | India | 29 | Female | B.1.617.2 | Second |
| hCoV-19/India/MH-ACTREC-153/2021 | betacoronavirus | EPI_ISL_2530379 | 29-04-2021 | India | 59 | Male | B.1.617.2 | Second |
| hCoV-19/India/MH-ACTREC-154/2021 | betacoronavirus | EPI_ISL_2530384 | 06-05-2021 | India | 26 | Female | B.1.617.2 | Second |
| hCoV-19/India/MH-ACTREC-155/2021 | betacoronavirus | EPI_ISL_2530388 | 18-05-2021 | India | 81 | Male | B.1.617.2 | Second |
| hCoV-19/India/MH-ACTREC-156/2021 | betacoronavirus | EPI_ISL_2530390 | 18-05-2021 | India | 69 | Female | B.1.617.2 | Second |
| hCoV-19/India/MH-ACTREC-157/2021 | betacoronavirus | EPI_ISL_2530391 | 28-05-2021 | India | 9 | Male | B.1.617.2 | Second |
| hCoV-19/India/MH-ACTREC-158/2021 | betacoronavirus | EPI_ISL_2530392 | 31-05-2021 | India | 61 | Male | B.1.617.2 | Second |
| hCoV-19/India/MH-ACTREC-159/2021 | betacoronavirus | EPI_ISL_2530394 | 01-06-2021 | India | 38 | Male | B.1.617.2 | Second |
