# Supplementary material for: Genomic Analysis of AZD1222 (ChAdOx1) Vaccine Breakthrough Infections in the City of Mumbai
Source: Int J Clin Pract. 2022 Feb 11;2022:2449068. doi: 10.1155/2022/2449068 (PMC9159196; doi:10.1155/2022/2449068)
Supplement: Supplementary Materials — Supplementary Table 1: details along with GISAID ID of all sequenced SARS-CoV-2 genomes in this study. Supplementary Table 2: distribution of severe COVID-19 among the unvaccinated (n = 92) patients according to clinical and genomic variables (continuous variables were dichotomized based on the median value in the entire cohort). Supplementary Table 3: distribution of severe COVID-19 among the vaccinated (n = 67) patients according to clinical and genomic variables (continuous variables were dichotomized based on the median value in the entire cohort). [file 2449068.f1.zip › 2449068.f1/Supplementary Table 2.docx]

**Supplementary Table 2: Distribution of severe COVID-19 among the unvaccinated (n=92) patients according to clinical and genomic variables (Continuous variables were dichotomized based on median value in the entire cohort).**

| **Category** | **Univariable Analysis** | | **Multivariable Analysis** | |
| --- | --- | --- | --- | --- |
|  | **% severe disease,**  **Odds Ratio (95% CI)** | **p-value** | **Odds Ratio (95% CI)** | **p-value** |
| **Age**  [≥36 (n=48) vs <36 (n=44) years] | 20.8% vs. 2.3%,  11.31 (1.38-92.54) | **0.008** | 9.44 (1.17-79.78) | **0.04** |
| **Gender**  [male (n=48) vs female (n=44)] | 14.6% vs. 9.1%,  1.71 (0.46-6.29) | 0.53 | 1.56 (0.39-6.20) | 0.53 |
| **Ct value of E gene**  [<22.5 (n=48) vs ≥22.5 (n=44)] | 12.5% vs. 11.4%,  1.11 (0.31-3.96) | 0.87 |  |  |
| **Ct value of N gene**  [<23 (n=47) vs ≥23 (n=45)] | 12.8% vs. 11.1%,  1.17 (0.33 to 4.14) | 0.81 |  |  |
| **Total no. of amino acid mutations per sample**  [≥22 (n=44) vs <22 (n=48)] | 13.6% vs. 10.4%,  1.36 (0.38 to 4.81) | 0.75 |  |  |
| **Diabetes**  [presence (n=8) vs absence (n=84)] | 25% vs. 10.7%,  2.78 (0.49-15.88) | 0.24 |  |  |
| **Hypertension**  [presence (n=7) vs absence (n=85)] | 14.3% vs. 11.7%,  1.25 (0.14-11.48) | 0.84 |  |  |
| **Malignancy**  [presence (n=27) vs absence (n=65)] | 22.2% vs. 7.7%,  3.43 (0.95-12.41) | 0.07 | 2.11 (0.54-8.17) | 0.28 |
| **Comorbidity**  [presence of any comorbidity (n=39) vs absence of all comorbidities (n=53)] | 17.9% vs. 7.5%,  2.67 (0.73-9.89) | 0.19 |  |  |
| **PANGOLIN Lineages** | | | | |
| **Kappa B.1.617.1**  [presence (n=40) vs absence (n=52)] | 12.5% vs. 11.5%,  1.09 (0.31-3.88) | 0.89 |  |  |
| **Delta B.1.617.2**  [presence (n=46) vs absence (n=46)] | 13.0% vs. 10.9%,  1.23 (0.35-4.35) | 0.75 | 1.28 (0.34-4.91) | 0.72 |
| **Spike Protein Mutations** | | | | |
| **D614G**  [presence (n=91) vs absence (n=1)] | 12.1% vs. 0.0%,  - (-) | 0.71 |  |  |
| **P681R**  [presence (n=90) vs absence (n=2)] | 12.2% vs. 0.0%,  - (-) | 0.59 |  |  |
| **L452R**  [presence (n=90) vs absence (n=2)] | 12.2% vs. 0.0%,  - (-) | 0.59 |  |  |
| **E484Q**  [presence (n=43) vs absence (n=49)] | 11.6% vs. 12.2%,  0.94 (0.27-3.34) | 0.93 |  |  |
| **T19R**  [presence (n=50) vs absence (n=42)] | 12.0% vs. 11.9%,  1.01 (0.28-3.58) | 0.99 |  |  |
| **R158G**  [presence (n=47) vs absence (n=45)] | 10.6% vs. 13.3%,  0.77 (0.22-2.74) | 0.76 |  |  |
| **T478K**  [presence (n=47) vs absence (n=45)] | 12.8% vs. 11.1%,  1.17 (0.33-4.14) | 0.81 |  |  |
| **G142D**  [presence (n=31) vs absence (n=61)] | 6.5% vs. 14.8%,  0.40 (0.08-1.97) | 0.32 |  |  |
| **T95I**  [presence (n=27) vs absence (n=65)] | 14.8% vs. 10.8%,  1.44 (0.38-5.39) | 0.73 |  |  |
| **A222V**  [presence (n=28) vs absence (n=64)] | 14.3% vs. 10.9%,  1.36 (0.36-5.07) | 0.73 |  |  |
| **Q1071H**  [presence (n=21) vs absence (n=71] | 14.3% vs. 11.3%,  1.31 (0.32-5.47) | 0.71 |  |  |
| **H1101D**  [presence (n=18) vs absence (n=74)] | 5.5% vs. 13.5%,  0.38 (0.05-3.15) | 0.69 |  |  |
| **D950N**  [presence (n=16) vs absence (n=76)] | 18.8% vs. 10.5%,  1.96 (0.46-8.39) | 0.39 |  |  |
| **E154K**  [presence (n=11) vs absence (n=81)] | 27.3% vs. 9.9%,  3.42 (0.75-15.56) | 0.12 |  |  |
| **Q677H**  [presence (n=6) vs absence (n=86)] | 16.7% vs. 11.6%, 1.52 (0.16-14.36) | 0.54 |  |  |
