# Supplementary material for: Genomic Analysis of AZD1222 (ChAdOx1) Vaccine Breakthrough Infections in the City of Mumbai
Source: Int J Clin Pract. 2022 Feb 11;2022:2449068. doi: 10.1155/2022/2449068 (PMC9159196; doi:10.1155/2022/2449068)
Supplement: Supplementary Materials — Supplementary Table 1: details along with GISAID ID of all sequenced SARS-CoV-2 genomes in this study. Supplementary Table 2: distribution of severe COVID-19 among the unvaccinated (n = 92) patients according to clinical and genomic variables (continuous variables were dichotomized based on the median value in the entire cohort). Supplementary Table 3: distribution of severe COVID-19 among the vaccinated (n = 67) patients according to clinical and genomic variables (continuous variables were dichotomized based on the median value in the entire cohort). [file 2449068.f1.zip › 2449068.f1/Supplementary Table 3.docx]

**Supplementary Table 3: Distribution of severe COVID-19 among the vaccinated (n=67) patients according to clinical and genomic variables (Continuous variables were dichotomized based on median value in the entire cohort).**

| **Category** | **Univariable Analysis** | |
| --- | --- | --- |
|  | **% severe disease,**  **Odds Ratio (95% CI)** | **p-value** |
| **Age**  [≥36 (n=32) vs <36 (n=35) years] | 6.3% vs. 2.9%,  2.27 (0.19-26.27) | 0.60 |
| **Gender**  [male (n=33) vs female (n=34)] | 6.1% vs. 2.9%,  2.13 (0.18-24.67) | 0.61 |
| **Ct value of E gene**  [<22.5 (n=31) vs ≥22.5 (n=36)] | 0.0% vs. 8.3%,  - (-) | 0.24 |
| **Ct value of N gene**  [<23 (n=33) vs ≥23 (n=34)] | 3.0% vs. 5.9%,  0.5 (0.04 to 5.79) | 0.57 |
| **Total no. of amino acid mutations per sample**  [≥22 (n=39) vs <22 (n=28)] | 2.6% vs. 7.1%,  0.34 (0.03 to 3.97) | 0.37 |
| **Diabetes**  [presence (n=6) vs absence (n=61)] | 0.0% vs. 4.9%,  - (-) | 0.57 |
| **Hypertension**  [presence (n=8) vs absence (n=59)] | 0.0% vs. 5.1%,  - (-) | 0.51 |
| **Malignancy**  [presence (n=2) vs absence (n=65)] | 0.0% vs. 4.6%,  - (-) | 0.76 |
| **Comorbidity**  [presence of any comorbidity (n=19) vs absence of all comorbidities (n=48)] | 0.0% vs. 6.25%,  - (-) | 0.26 |
| **Kappa B.1.617.1**  [presence (n=37) vs absence (n=30)] | 0.0% vs. 10.0%,  - (-) | **0.05** |
| **Delta B.1.617.2**  [presence (n=28) vs absence (n=39)] | 10.7% vs. 0.0%,  - (-) | **0.04** |
| **D614G**  [presence (n=67) vs absence (n=0)] | 4.5% vs. -  - (-) | - |
| **P681R**  [presence (n=67) vs absence (n=0)] | 4.5% vs. -  - (-) | - |
| **L452R**  [presence (n=67) vs absence (n=0)] | 4.5% vs. -  - (-) | - |
| **E484Q**  [presence (n=37) vs absence (n=30)] | 0.0% vs. 10.0%,  - (-) | **0.05** |
| **T19R**  [presence (n=29) vs absence (n=38)] | 10.3% vs. 0.0%,  - (-) | **0.04** |
| **R158G**  [presence (n=29) vs absence (n=38)] | 10.3% vs. 0.0%,  - (-) | **0.04** |
| **T478K**  [presence (n=29) vs absence (n=38)] | 10.3% vs. 0.0%,  - (-) | **0.04** |
| **G142D**  [presence (n=23) vs absence (n=44)] | 0.0% vs. 6.8%,  - (-) | 0.20 |
| **T95I**  [presence (n=22) vs absence (n=45)] | 0.0% vs. 6.7%,  - (-) | 0.22 |
| **A222V**  [presence (n=13) vs absence (n=54)] | 23.1% vs. 0.0%,  - (-) | **0.006** |
| **Q1071H**  [presence (n=17) vs absence (n=50)] | 0.0% vs. 6.0%,  - (-) | 0.30 |
| **H1101D**  [presence (n=15) vs absence (n=52)] | 0.0% vs. 5.8%,  - (-) | 0.34 |
| **D950N**  [presence (n=10) vs absence (n=57)] | 0.0% vs. 5.3%,  - (-) | 0.46 |
| **E154K**  [presence (n=13) vs absence (n=54)] | 0.0% vs. 5.5%,  - (-) | 0.38 |
| **Q677H**  [presence (n=4) vs absence (n=63)] | 25.0% vs. 3.2%,  10.17 (0.71-146.17) | **0.04** |
